# The CABANA model 2017–2022: research and training synergy to facilitate bioinformatics applications in Latin America

**DOI:** 10.3389/feduc.2024.1358620

**Published:** 2024-07-04

**Authors:** Rebeca Campos-Sánchez, Ian Willis, Piraveen Gopalasingam, Daniel López-Juárez, Marco Cristancho, Cath Brooksbank

**Affiliations:** 1 Centro de Investigación en Biología Celular y Molecular, Universidad de Costa Rica, San José, Costa Rica; 2 EMBL’s European Bioinformatics Institute (EMBL-EBI), Hinxton, United Kingdom; 3 Plant Pathology Department, National Coffee Research Center, Manizales, Colombia

**Keywords:** bioinformatics, Latin America, training, research, diversity equity and inclusion

## Abstract

The CABANA project (Capacity Building for Bioinformatics in Latin America) was funded by the UK’s Global Challenges Research Fund in 2017 with the aim to strengthen the bioinformatics capacity and extend its applications in Latin America focused on three challenge areas – communicable diseases, sustainable food production and protection of biodiversity. For 5 years, the project executed activities including data analysis workshops, train-the-trainer workshops, secondments, eLearning development, knowledge exchange meetings, and research projects in 10 countries. The project was successful in accomplishing all its goals with a major impact on the region. It became a model by which the research needs determined the training that was delivered. Multiple publications and over 800 trainees are part of the legacy of the project.

## 1 Introduction

Bioinformatics has now become essential to accelerate scientific discoveries and applications with important implications for human health, food security, and biodiversity protection. Recent examples include the SARS-CoV-2 pandemic (Maxwell et al., 2023), and one-health approaches to antimicrobial resistance (Bravo et al., 2023).

Latin America (LatAm) is a major global producer of food, a region with unparalleled biodiversity, and has made significant progress toward reducing its burden of communicable diseases. Although media coverage may suggest otherwise, sound governance and the development of civil society continue to grow. LatAm is poised to contribute equitably to scientific endeavors and to share its learning with less developed regions. For example, the region has a major stake in biodiversity protection owing to six of the world’s 17 megadiverse countries being in the region; it has immense human diversity, and it has a tremendous wealth of food sources, ranging from widely exported staples to wild races of crops and varieties used only by isolated populations living in specific ecological niches (OECD, Commission, E., America, C. D. B. of L., America, E. C. for L., and Caribbean, the, 2022). Targeted support for multilateral, international scientific collaborations focused on molecular biodata would contribute towards giving LatAm the voice it needs to contribute equitably to addressing pressing global challenges.

The CABANA project (Capacity Building for Bioinformatics in Latin America) was set up with the goal of strengthening the region’s biodata science capacity. CABANA was initially conceived in the autumn of 2016, in a Skype call between Marco Cristancho, then at BIOS (Colombian Bioinformatics and Computational Biology Center) in Colombia, and Cath Brooksbank at EMBL-EBI (European Molecular Biology Laboratory - European Bioinformatics Institute). The previous year, two EMBL-EBI scientists, Leyla Garcia and Emily Perry, had delivered training at BIOS. Previous to this, EMBL-EBI had also benefited from a Biotechnology and Biological Sciences Research Council (BBSRC) Partnership Award with Brazil, as part of a livestock genomics project led by the Roslin Institute (UK Research and Innovation, 2013); this had enabled scientists from five Brazilian states to co-deliver bioinformatics training, including train-the-trainer (TtT) activities across Brazil. Marco and Cath, excited by the prospect of putting together a pan-LatAm network for bioinformatics training in response to the formation of the UK-led Global Challenges Research Fund (GCRF), rapidly convened a list of contacts across six LatAm countries, including expertise in food security, biodiversity, and communicable diseases.

The GCRF was formed in response to a UK Government decision to increase its contribution to the Official Development Aid (ODA) budget from 0.5 to 0.7% of Gross National Income (GNI). The funding calls launched by GCRF, managed by the newly formed UK Research and Innovation (UKRI), were something of a departure from classical research funding calls; they required close linkage to the United Nations Sustainable Development Goals (UN-SDG) (United Nations, 2015), and governance methods that are (or were) more typically adopted by international development projects than research projects. A consortium of ten partners, nine from LatAm and one based in the United Kingdom, was rapidly convened.

The purpose of CABANA was to accelerate the implementation of data-driven biology in the region by creating a sustainable capacity-building program focusing on three challenge areas – communicable diseases, sustainable food production, and protection of biodiversity. Our decision to focus on three areas of sustainable development across a huge geographical area put us in stark contrast to other successful applicants for the GCRF-GROW awards (there were 37 in total); many focused on a single challenge in a single country. Nevertheless, our broad-brush approach has proved to be flexible and effective, and all our efforts are united by open science and inclusivity.

## 2 Materials and methods

**Figure 1:**
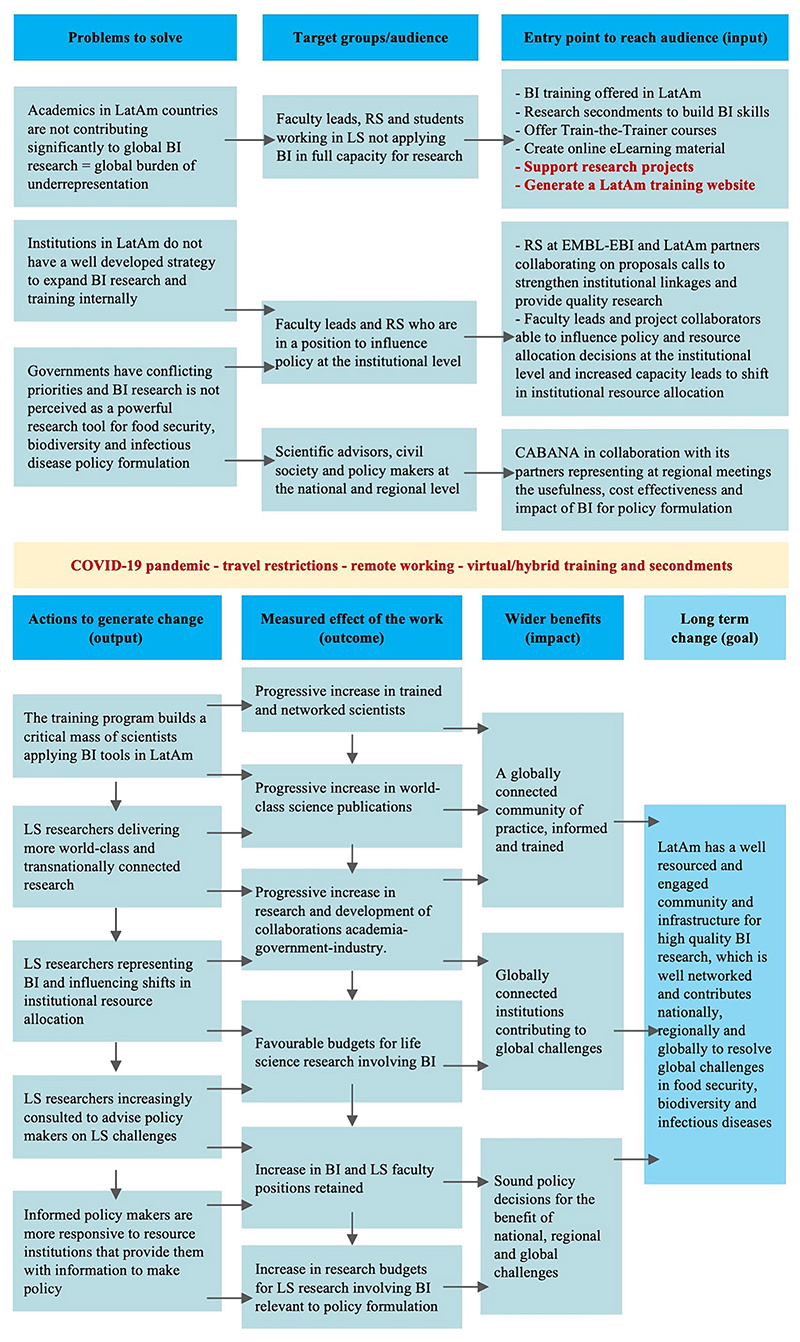
Theory of change of CABANA. The project focused on three problems to solve in LatAm. The major target groups that can make a change are faculty, research scientists, and students. We proposed five actions to generate change with global benefits. The red text highlights the amendments made to the project during the COVID-19 pandemic. BI, Bioinformatics; LatAm, Latin America; LS, Life Sciences; RS, Research Scientists.

The CABANA project began on 1st October 2017, and in November 2017 the CABANA co-investigators met for the first time in Campinas, Brazil for a kick-off workshop that involved identifying and analyzing problems and bottlenecks common to all the partners, and planning how CABANA would address those bottlenecks. A logical framework (Supplementary Data S1) was developed from that meeting and has been central to the evolution and current model of the project. A theory of change model (Figure 1) was developed as a requirement for the GCRF grant application to delineate the steps needed to obtain long-term change (Reinholz and Andrews, 2020). These steps were revised during the execution of the project. Our intended impact was that, by the end of the project, a pan-LatAm and sustainable capacity-building program for bioinformatics research would be operating with at least six partner institutions. After 5 years of execution, the model has proved to be adaptable and relevant to biodata science in the LatAm region, with major signature activities and outcomes that we present in this manuscript. Currently, the project is funded by the Chan Zuckerberg Initiative (CZI, grant number 2022–316296). It has become CABANAnet, a network of institutions and researchers based in LatAm joined by common research interests, including the application of bioinformatics, and now led by LatAm partners.

The CABANA project is a program that strengthens individual, institutional, and regional capacity through six main activities: secondments (long-term visits and exchanges), train-the-trainer activities, training workshops, eLearning, research projects, and knowledge exchange meetings (KEM) (Table 1). All of these activities represent opportunities to train early-stage biodata scientists and to foster transnational collaborations – within and beyond Latin America. Moreover, these activities have proved helpful in many contexts (Wright et al., 2010; Brazas and Ouellette, 2013; Via et al., 2013, 2019; Kolympiris et al., 2019; McGrath et al., 2019; da Silva et al., 2021).

**Figure 2:**
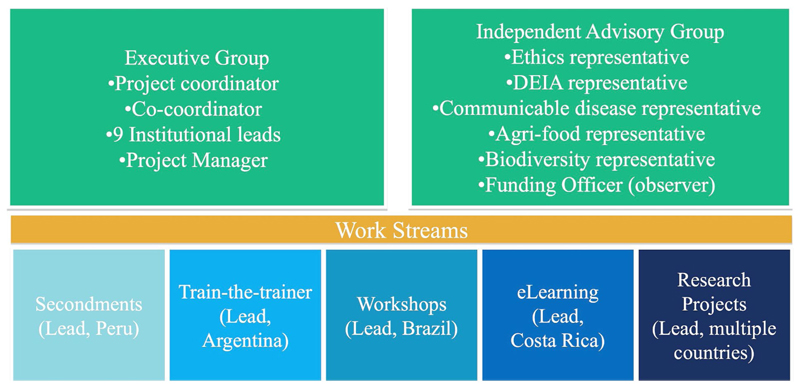
CABANA’s governance structure 2017–2022. The executive group led the project, and the advisory group made suggestions once a year based on the annual report of the project. Every workstream was led by a partner in different countries.

CABANA’s governance structure was, and remains, simple and equitable (Figure 2). The structure included an executive board with representation from every partner group; an independent advisory board with coverage of all our challenge areas and important cross-cutting topics such as Diversity, Equity, Inclusion, and Accessibility (DEIA) and ethics; and a set of workstreams, each with a lead from a different country.

Every month the executive board met to evaluate the progress and to plan for the months ahead. Additionally, every year the entire consortium met in person (or virtually during the COVID-19 pandemic) for the All-hands meeting to celebrate achievements, assess progress, and plan for long-term planning (for example, sustainability planning). For part of the All-hands meeting, the advisory board joined the meeting, to evaluate progress objectively and provide independent steer for the following year. Once the consortium had received feedback from the advisory board, the final day of the all-hands meeting was used to consider the advisory board input and incorporate it into CABANA’s plans. These strategies proved to be effective and responsive. The advisory board was international, with long experience in their field and acquainted with the LatAm region, which allowed them to provide insightful ideas and solutions. Although funding did not allow the participation of all the secondees in every meeting, we ensured representative participation; three of our original secondees are now representing their institutions as partners of CABANAnet (including CABANAnet’s principal investigator). New partners have been added to CABANAnet and the addition of further partners is planned.

The progress and outcomes of the project have been measured quantitatively and qualitatively every year from 2017–2022 using two tools: the baseline questionnaire, and a monitoring and evaluation questionnaire (M&E, Supplementary Data S2), which triangulates with project activities. Both questionnaires were identical, the difference was the time when they were used. The baseline questionnaire was used to set the base numbers of the project. The M&E was completed every year; the findings were reviewed annually and discussed at the All-hands meeting and the advisory board meeting.

The questionnaires provided information about the state of bioinformatics training in the region, the availability of bioinformatics posts for trained personnel, and the level of international and regional research using bioinformatics. Moreover, it addressed the level of engagement of scientists in the region promoting the use of computational biology to inform policy and policymakers. The baseline also gauged the current level of regional collaborations and opportunities for secondments of researchers between the partner institutions in the process.

The M&E questionnaire evaluated the delivery of the project objectives against the baseline questionnaire. The same M&E questionnaire was sent to the partners each year at the same time to track progress against project objectives and goals; it informed progress and areas needing more attention.

The baseline and M&E reports provided the project with a mix of qualitative and quantitative information, which enabled the project team to triangulate and provide tangible evidence of the progress against the project’s activities, objectives, and goals. Progress was tracked against the objectives set in the logical framework. The reports were used to inform our annual All-hands meeting and provided evidence for the advisory board and UKRI of the consortium’s progress in meeting its objective of a sustainable capacity-building program for bioinformatics in the region. The partners continue to complete an M&E questionnaire each year to inform progress and areas needing further attention and have recently revised it whilst retaining sufficient continuity to monitor progress over CABANA’s entire lifespan.

For the project activities (Table 1), we measured multiple aspects. The secondment statistics included the nationality and gender of the secondees. After completion of their secondment, we also monitored the involvement of each secondee with CABANA and their institutions. Train-the-trainer (TtT) and workshops were evaluated by the participants after each event. We kept track of nationality, gender, research themes, and career progression to evaluate the impact of these programs. The reach and impact of eLearning materials were measured using download statistics from the F1000 CABANA collection^1^, and the use of the platform at cabana.online.

From the year 2020 and onwards, the partners also developed seven cross-border research projects. Multiple routes to impact were measured including report submissions and presentation of findings at regional conferences. The research teams recruited their own secondees to contribute to the research.

Secondment and research impact were also measured by the number of projects that deposited genomics data in databases, particularly the European Nucleotide Archive (ENA) as all the projects had nucleotide sequence data as a significant output. Additionally, we kept track of the number of publications by CABANA partners where the project was involved and cited. The titles and abstracts of 39 publications were used to generate a word cloud to determine the main areas of impact.

## 3 Results

Here we present the summary of five years of the project execution under the GCRF funds, from 2017 to 2022. The project is currently funded by CZI and has been rebranded as CABANAnet^2^.

### 3.1 Main outcomes and impact

**Figure 3:**
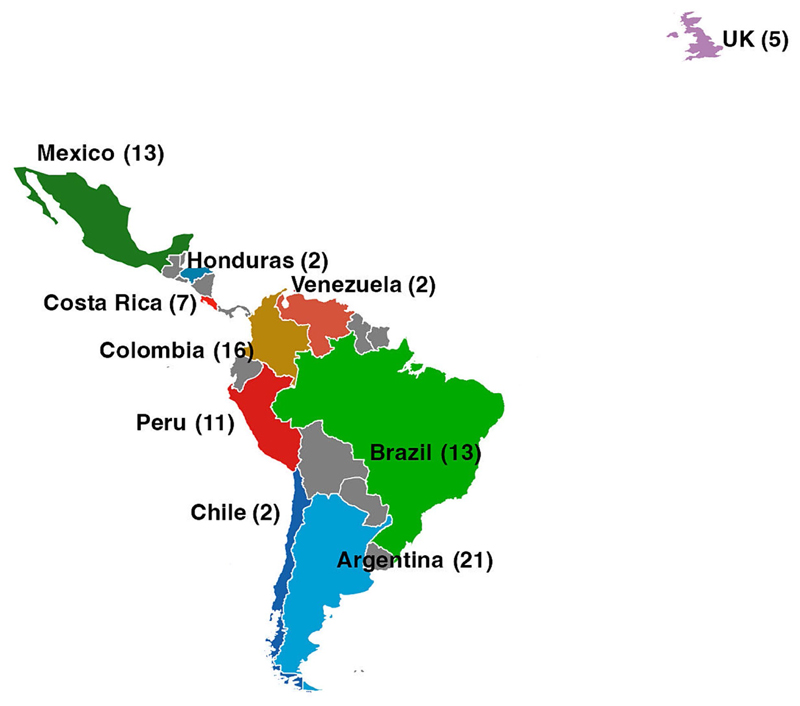
LatAm region and United Kingdom map showing the number of CABANA activities by location performed virtually or in person, this includes workshops, secondments, and Train-the-Trainer. Secondments are counted by the secondee country of origin, and not the host institution.

In summary, the project implemented activities in 10 LatAm countries, but the selected trainees or participants came from 15 countries (Figure 3). This is a significant representation of LatAm and the Caribbean, which is conformed by 33 countries.

**Figure 4:**
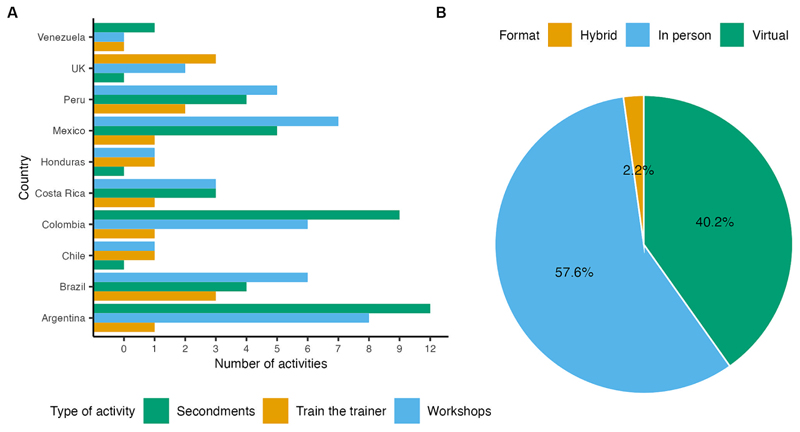
Basic statistics of CABANA activities. **(A)** Number of secondments (S), Train-the-trainer (TtT) and workshops (Ws) delivered by country. **(B)** Modality type of all the activities. Some secondments and workshops were delivered virtually due to COVID-19 restrictions on travel, though initially all activities were planned to be in-person.

The project surpassed the goals for the Secondments (S) and Workshops (Ws) activities, explained below, reaching three countries in addition to those hosting the original partners, which included Chile, Honduras, and Venezuela (Figure 4A). Most of these activities benefited participants from the partner countries as planned in the project. Fifty-two percent of the activities were delivered in person (Figure 4B), and the virtual activities were planned in response to the SARS-CoV-2 restrictions, but they were equally effective as the in-person versions.

**Figure 5:**
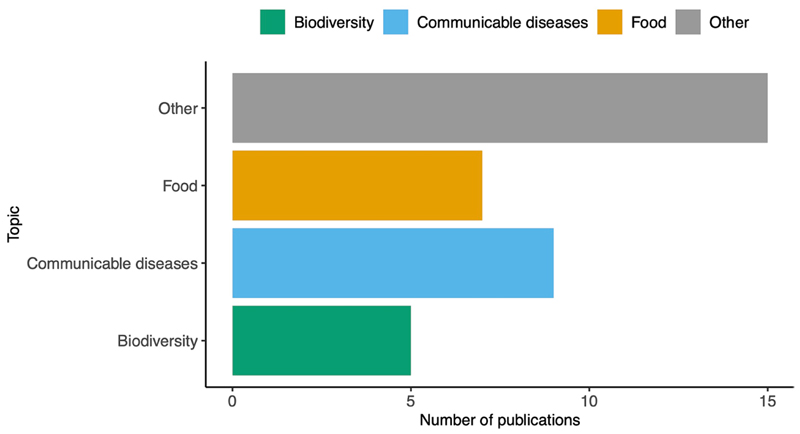
Number of publications attributed to CABANA distributed by topic. The list includes eLearning tutorials and scientific publications. The year of publication ranges from 2017 to 2023.

**Figure 6:**
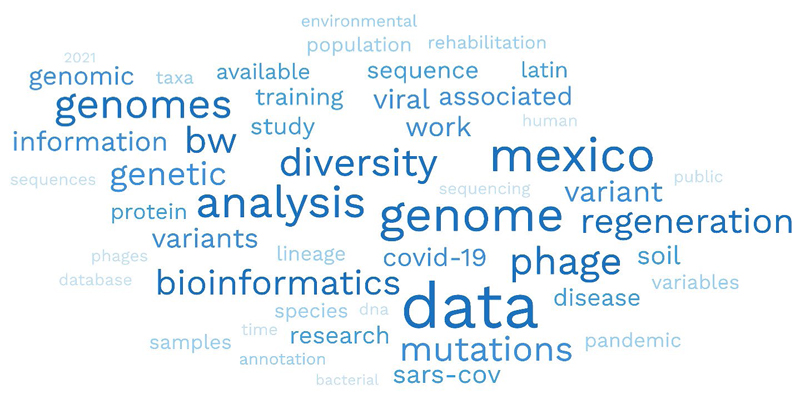
Word cloud of the titles and abstracts of 39 CABANA publications. The figure includes the main 50 words. For scale purposes, the word data was used 36 times.

Forty-seven publications were attributed to the network activities and research, including the eLearning material published in the F1000 CABANA collection (Supplementary Data S3). Of these, 36 are searchable in Europe PMC using the grant number (Medina-Castellanos et al., 2018; Radusky et al., 2018; Amann et al., 2019; Darnet et al., 2019; Holguín et al., 2019; Lemos et al., 2019; Papatheodorou et al., 2019; Filippi et al., 2020; Gastauer et al., 2020; Hounkpe et al., 2020; Hufsky et al., 2020; Ochoa et al., 2020; Rocha et al., 2020; Scott et al., 2020; Ayala-Usma et al., 2021; Camarillo-Guerrero et al., 2021; Canto et al., 2021; Contreras-Moreira et al., 2021; Dillon et al., 2021; Matosinho et al., 2021; Moore et al., 2021; Silveira et al., 2021; Yates et al., 2021; Payaslian et al., 2021a, b; Atriztán-Hernández and Herrera-Estrella, 2022; Cabrera-Toledo et al., 2022; Laux et al., 2022; Merino et al., 2022; Zárate et al., 2022a,b; Loza et al., 2023; Molina-Mora et al., 2023; Rangel-Pineros et al., 2023; Sriraja et al., 2023; Lacarrubba-Flores et al., 2024). When classified by the three themes of CABANA, we found that communicable diseases dominated; we published five manuscripts on SARS-CoV-2 in LatAm (Figure 5). Other manuscripts included human genetics, data modeling, databases, training topics, and evolution studies. Some of these terms are evidenced in the word cloud (Figure 6) of the titles and abstracts of 39 publications. In addition, we observed the terms data, genome, Mexico, analysis, diversity, and BW (a SARS-CoV-2 variant) as the most used words, reflecting the focus of some of the manuscripts related to the project. Additionally, at least 28 open resources, ranging from nucleotide sequence datasets to open-access tutorials, were generated.

CABANA is now one of several LatAm efforts to stimulate biodata science in the region. While untangling cause and effect is near impossible, CABANA has contributed to a changing research culture that strongly values global equity and open science. Other pan-LatAm biodata science initiatives with similar values include LatinGenomes (2020), a project to enrich knowledge of human variation in the region, and Latinbiota (Martins, 2021), a pan-LatAm initiative to capture and understand information about the microbiome of LatAm’s diverse population and its impact on health. CABANA has also proactively reached out to regional and international initiatives with a bioinformati.cs training and knowledge-exchange remit; these include the International Society of Computational Biology (ISCB^3^ the world’s leading professional body for bioinformaticians) and its tremendously proactive regional student groups (Shome et al., 2019); SoiBio, the Iberoamerican Bioinformatics Society^4^, several national societies (Argentina, Brazil, Mexico, Colombia and most recently Peru) and, since its inception during the COVID-19 pandemic, Metadocencia^5^. Through CABANA, new representatives from LatAm on the Board of Directors of the ISCB were included. CABANAnet also has representation on the ISCB’s education committee, and the 2024 ISCB LatAm conference was co-organized by CABANAnet partners.

### 3.2 Secondments

Thsix CABANA project had a target of achieving 28 research secondments in the UK and LatAm. The project exceeded its objective by achieving 39 secondments in three calls for secondees in 2018, 2019, and 2020. The secondment duration was typically 6 months, but part of the second cohort remained longer in the UK because of travel restrictions precipitated by the COVID-19 pandemic.

In the first two calls for secondees, 20 secondments were completed in the UK, 11 in the first round and nine in the second round, of which 19 were at EMBL-EBI and one at The Earlham Institute, Norwich. In the second call, the project had two secondments in LatAm. The two secondees were from institutions in Mexico who visited the Universidad de San Martín de Porres, Lima, Peru, with Professor Ricardo Fujita and The University of Buenos Aires with Professor Adrian Turjanski.

The secondment program was interrupted by the onset of the COVID-19 pandemic. Travel between LatAm and the UK was not possible, and many institutions did not allow travel or visiting scholars. The CABANA team therefore explored the possibility of a LatAm call for secondments. The aim was to assign secondees to a specific CABANA Innovation Award research project supervised by a CABANA PI (Principal Investigator), enabling them to contribute to the research and improve their bioinformatics skills, primarily whilst working from home. The virtual nature of training during the pandemic reduced training costs considerably, lowered the carbon footprint, and provided a more sustainable method for providing training opportunities in a resource-poor environment.

Although conventional secondments with a visit to a new institution’s laboratory and mentoring from a new mentor remain ideal, an important component of the secondment program is the creation of long-term collaborative relationships between the secondee and supervisor. The evidence suggests that these relationships are developed equally well with virtual vs. in-person secondments (measured as the number of publications, collaborations, and applications), and opportunities for additional studies and exchange visits may ensue beyond the project. The mix of virtual and conventional secondment programs offers an alternative and effective model for a sustainable future.

The third cohort comprised 17 secondments, all based in LatAm. Fourteen of these were supervised by the CABANA partners in virtual settings and worked on the Innovation Awards supported by the project.

The provenance of the secondees was four from Brazil, 12 from Argentina, five from Mexico, nine from Colombia, three from Costa Rica, four from Peru, and two from Venezuela (Figure 4). We achieved a perfect gender balance in this program (50% male, 50% female). Of the 39 secondees, 28 stated they had submitted their data to publicly available databases or were confident they would do so. Towards the latter end of the project and the COVID-19 travel restrictions, the secondment program was wholly managed from LatAm.

### 3.3 Train the trainer

CABANA had a target of completing four in-depth TtT bioinformatics workshops at EMBL-EBI in the UK. The objective for the participants was to undergo a one-week-long in-depth training and then apply their new skills as trainers in an EMBL-EBI course related to one of CABANA’s challenge areas. After this course, the new trainers would then reconvene to discuss how they would apply their new skills in their own context. The method of training evolved during the project as it became clear that the cost and duration of the in-depth workshops were limiting participation. During the pandemic, travel restrictions also became a factor.

One in-depth TtT was held in the initially proposed format at EMBL-EBI. From then on, the program developed a two-part virtual approach comprising a 2 to 3-day interactive, virtual TtT workshop followed by an advanced virtual scientific workshop; trainees participating in the virtual TtT were encouraged to present parts of the virtual bioinformatics workshop. In March 2020, to reinforce capacity strengthening, it was agreed that activities should be managed by CABANA’s LatAm partners.

The project output of four in-depth TtT activities was therefore met. This approach enabled CABANA to support the development of bioinformatics trainers in each of our UN SDG-linked challenge areas:

- Food Security (livestock genomics, hosted by EMBL-EBI, UK, in-person)
- Food Security (Crop plant genomics, hosted by Universidad de Costa Rica-UCR, Costa Rica, virtual)
- Infectious Diseases (hosted by Universidad Peruana Cayetano Heredia – UPCH and Universidad San Martin de Porres – USMP, Peru, virtual)
- Protection of biodiversity (hosted by Universidade Federal de Minas Gerais – UFMG and Instituto Tecnologico Vale – ITV, Brazil, virtual)

In addition, ten short, foundational TtT workshops were conducted – these events coincided with other CABANA meetings or national or international bioinformatics conferences, including ISCB Latin America 2018 and 2020, XMXP20, and A2B2C conference 2021 (Supplementary Data S4). To date, through these short workshops, we have trained 111 (38 females, 36 males, 37 no data) LatAm scientists to deliver training, of which 28 (~25% total, 16 females and 12 males) have delivered training for CABANA-related activities.

Six secondees took their training to the next level – independently organizing and delivering Train the Trainer courses in Portuguese, Spanish, and/or English. One example is the in-depth ‘Train-the-lecturer’ activity that was developed by Patricia Carvajal-Lopez, as part of her secondment project. This was delivered virtually to a group of 10 lecturers at Universidad Nacional de Agricultura in Honduras over a period of 7 weeks with input from two other CABANA secondees (María Bernardi and Mindy Muñoz). An observer-to-trainer model was also implemented to maximize capacity strengthening. This is a model for training life-science lecturers in fundamental bioinformatics at sufficient depth to support its appropriate incorporation into undergraduate courses.

As discussed earlier, an important component of the project’s sustainability was to ensure that trainees and secondees remained well-connected with PIs at the partner institutions and build long-term relationships. The project team has endeavored to foster these relationships by inviting secondees and trainees to participate in the All-hands meetings and planning workshops.

### 3.4 Workshops

CABANA aimed to run 28 bioinformatics workshops in LatAm (Supplementary Data S5). The project reached its objective, running 28 workshops for LatAm scientists, providing training for over 800 people across the region, with 141 travel fellowships awarded to encourage attendance from underrepresented genders and ethnicities (see section 3.8 Diversity, Equity, Inclusion, and Accessibility). Owing to the risks posed by the pandemic, from March 2020 to the end of the UKRI-funded CABANA project, all workshops were virtual. However, in-person workshops have now resumed as part of the CABANAnet award, with the most recent one being hosted at the University of Costa Rica and led by one of CABANA’s first cohort of secondees, Valeria Faggioli.

**Figure 7:**
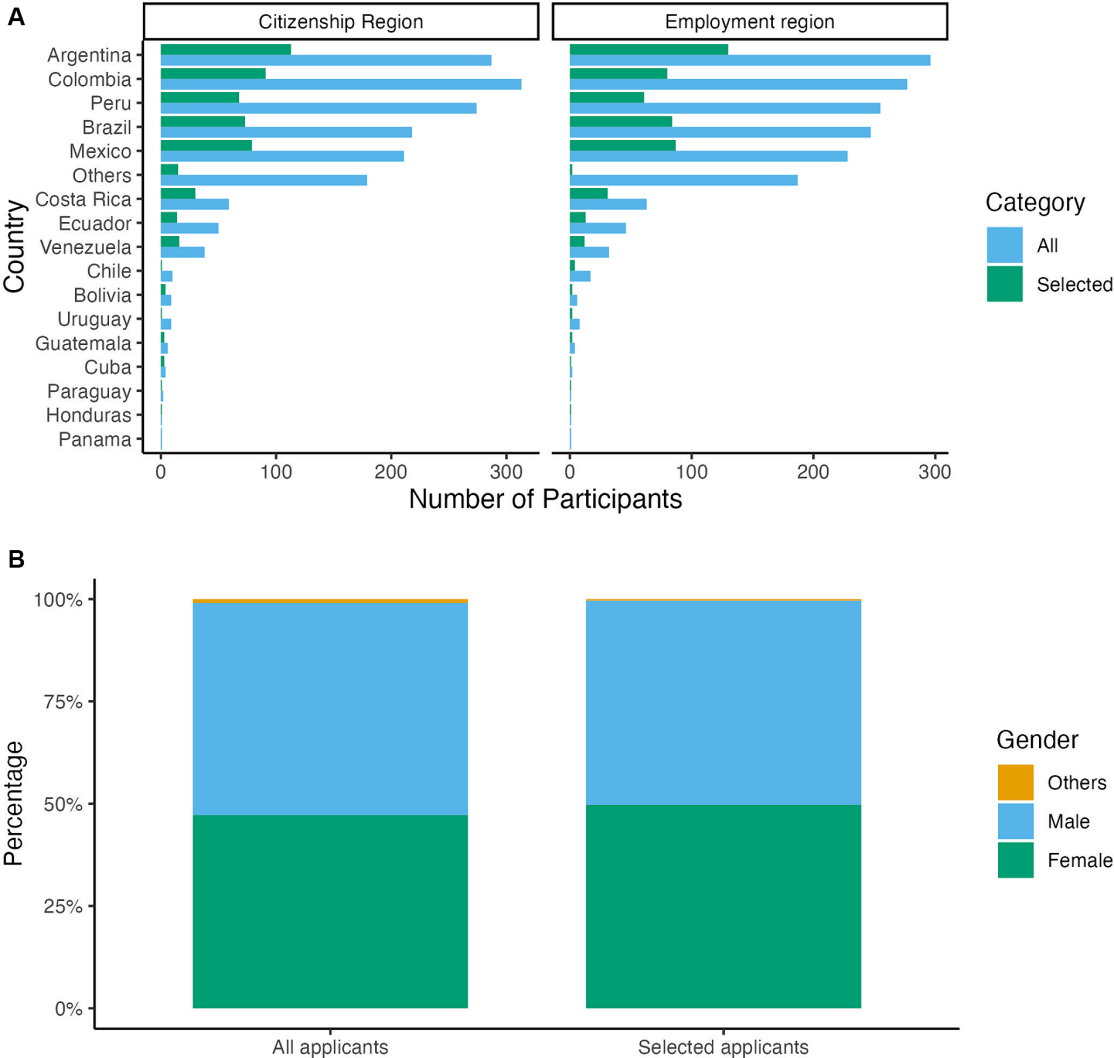
Participants’ data from 20 CABANA workshops. **(A)** Citizenship and employment region of all applications and selected participants show the effect of the country where the workshop took place. **(B)** The gender divided by all applications and selected participants provide evidence of the gender balance in this type of activity.

For 20 of the workshops, we received applications from 17 countries in LatAm and the Caribbean; most of the applicants also worked in their country of citizenship (Figure 7A). This is relevant since the impact of the training can be measured locally. We also received applications from other regions, including Africa, Asia, Europe, North America (Canada, USA, and Puerto Rico), the Caribbean, and Oceania, although we were explicit that only scientists working in LatAm and the Caribbean were eligible. A great proportion of the applications and selected participants came from the most highly populated countries – Argentina, Brazil, Colombia, Mexico, and Peru, which were also partners of CABANA. Costa Rica was also among the partners but provided fewer applications compared to larger countries. We also selected participants from Bolivia, Chile, Cuba, Ecuador, Guatemala, Honduras, and Paraguay, expanding the impact of training in the LatAm region. In terms of gender, we received 51.8% male, 47.2% female, and 1% non-binary or prefer-not-to-say applications (Figure 7B). For the selection process, we kept an even gender balance (Figure 7B).

Workshops were initially designed based on modular EMBL-EBI offsite events and were soon expanded to address bioinformatics skills gaps across different disciplines. Throughout the project, internal processes critical for workshop organization were improved based on organizer feedback. Processes were flexible to allow organizers to use existing event organization knowledge and receive support in areas such as ensuring a fair selection of trainees, awarding travel fellowships, and collecting feedback.

Workshop trainers were initially sourced from EMBL-EBI service teams, CABANA partners, and their professional networks. As the project progressed, CABANA secondees at EMBL-EBI were encouraged to take train-the-trainer workshops, and thereafter were given opportunities to deliver training sessions at workshops. Secondees embedded in service teams received in-depth training in EMBL-EBI resources and became trainers for these resources when they returned to their home institutions; some have also continued to lead workshop organization at their institutions. Through research secondments, LatAm training capacity in EMBL-EBI resources has markedly increased. This supports CABANA’s goals to increase bioinformatics use and data submission in the region.

Most workshop topics focused on genomics or metagenomics across the three challenge areas (Supplementary Data S5). Our LatAm partners have expertise in biodiversity and crop security, which was reflected in the workshop topics and continues to be so today. Workshops on communicable diseases were more difficult to organize owing to the disproportionately low representation of partners with expertise in this area; however, this was addressed in the second half of the CABANA project. Communicable disease workshops were organized by collaborators, including FIOCRUZ and the University of São Paulo (USP) in Brazil, and by secondees with expertise in communicable diseases, such as a workshop organized at Universidad de Antioquia in Colombia. The COVID-19 pandemic hastened the organization of workshops to disseminate viral genomics and surveillance knowledge across the region.

LatAm secondees and partners also supported the organization and delivery of workshops in Spanish. This allowed a wider pool of trainees to attend and benefit from high-quality bioinformatics training, and for training materials to be produced and made freely available in Spanish in line with UNESCO’s guidelines on Open Educational Resources.

### 3.5 eLearning

The initial target of the CABANA project was to establish nine new eLearning courses. The project activities were revised on the fly so that the entire eLearning component of the project was managed and created by our LatAm partners, led by The University of Costa Rica and the University of the Andes (UniAndes). We made public four ‘Essentials’ and five ‘Practical’ courses in the CABANA eLearning platform^6^ and in the CABANA F1000 Collection. The topics included genomics, transcriptomics, and metagenomics (parts I and II). In addition, we are migrating from the cabana.online/elearning platform to a new system hosted at University of Costa Rica (UCR). Students can register on the new platform, undergo the evaluation process, and upon successful program completion, they will receive a certificate from UCR. Part of the impact of this work is measured by the views and downloads from the F1000 CABANA collection (Table 2), and a growth in the number of users of the cabana.online/elearning platform reaching a total of 439 users by November 2023.

The Master’s course in Computational Biology at UniAndes has used several of these tutorials in its classes. Furthermore, several students at both UniAndes and UCR have independently used the CABANA tutorials to learn the pipelines featured in the ‘Essentials’ and ‘Practicals’ courses. In 2021, (August–December semester), UCR used the published tutorials in its Bioinformatics Master’s program (11 students). Incorporation of these materials into pre-existing graduate courses in the region is one way of ensuring that the materials will continue to be used and updated.

The MSc program in Computational Biology at UniAndes has been and continues to be supported by CABANA and EMBL-EBI each year. This course includes a module on online resources and biological data analysis, which covers at least 10 EMBL-EBI resources throughout 16 weeks. Each week, the students are requested to watch webinars available on EMBL-EBI’s ‘on-demand’ channel before participating in an interactive, live session with a trainer, where they take a deeper look at the resource and resolve any questions. Additionally, students are asked to combine different resources to build their own workflow or pipeline to answer a research question of their choosing. In the first 2 years of the project, the majority of the trainers were from EMBL-EBI. The 2020 and 2021 iterations of the module were modified so that the majority of the trainers were CABANA secondees. This opened the opportunity for the students to get a more direct experience of the types of applied research that can be done by using the different tools; additionally, in many cases, the training was delivered in Spanish, increasing comprehension and learning. The current setting has paved the way for students from other universities to join live. In 2021, 21 participants from Costa Rica (including undergraduate and graduate students) attended the live training sessions and completed the course to receive a participation certificate from UCR. In 2022, we had 35 applications from five LatAm countries, 22 were invited to participate, but only three completed the course. In 2023, due to an increased communications strategy, we had 203 applications from 12 LatAm countries (plus Portugal and USA); of the 35 selected participants 28 confirmed participation but the mean number of attendees from this cohort had reduced to eight by the end of the semester. The drop in online participation requires further evaluation. We hope to adapt the teaching methods that CABANA has used to create modules at other universities or to incorporate additional resources developed in LatAm.

### 3.6 Knowledge exchange meeting and external interactions

To sustain bioinformatics in the region, CABANA provided opportunities for the partners to participate in meetings with funders and policymakers. These meetings advocate for the importance of science in the region for addressing global challenges, thereby providing evidence of the need for adequate funding, staffing, and other resources, including computing infrastructure. The project tracked the number of policy meetings attended, the number of positions in bioinformatics, the sharing of data, and the increase in national and international collaborations.

The CABANA project identified early on that to create sustainable capacity strengthening at the level of individuals, organizations, and the region as a whole, more engagement with policymakers in LatAm was necessary. The aim was for our partners to become “key informants” by sharing research findings and the value of bioinformatics with policymakers and other stakeholders. The partners demonstrated an increase in engagement with key policymakers at meetings. Our conference program was restricted in 2020 and 2021 owing to the COVID-19 pandemic; nevertheless, three live conferences were delivered: two hybrid meetings were hosted in Mexico, one covering COVID-19 preparedness in the region and the other covering food security. There was also a closing conference in Colombia, attended by key regional funding bodies and networks, to discuss the project’s response to its three Global Challenges: communicable diseases, food security, and protection of biodiversity.

On average, an additional 10 regional policy meetings per year were attended by members of the CABANA consortium to advocate for more resources for bioinformatics. Thirty-nine meetings were held in 2019, 34 meetings were held in 2020, and 27 meetings in 2021. We noted a decrease in policy meetings towards the end of the project, which we attribute to the COVID-19 pandemic.

We have observed a noteworthy surge in international collaborations at our partner institutions. While our initial target was set at seven collaborations, our partners have actively engaged in 21 collaborative initiatives, data provided on the feedback from our baseline and M&E questionnaire.

### 3.7 Cross-border research

One of the capacity constraints identified by the CABANA team early in the project was the unavailability of funds for LatAm researchers to conduct research across the region. We also identified that LatAm research institutions were not proportionally represented in global bioinformatics consortia, where often the research leadership and funding emanates from Europe, the USA, or Canada. This dearth of participation has several knock-on effects, including limited global impact, poor regional representation at international conferences, limited mechanisms for regional cross-border collaboration, high costs of sequencing equipment and reagents, low efficiency of sequencing and analysis pipelines, skepticism over the benefits of data sharing, and lack of opportunities for skills development. These all represent barriers to global research collaboration and limit the ultimate benefit of research to humankind.

To stimulate regional science and representation, the CABANA project requested a budget amendment to allow calls for proposals in regional research and training capacity infrastructure. CABANA selected seven research projects that were instrumental in creating cohesiveness between the other four project workstreams (Supplementary Data S6). The data generated by these research projects is being used in training programs in LatAm. The project’s secondee program, which could no longer allow secondees to travel to the UK owing to the COVID-19 pandemic, was able to offer hybrid and/or virtual placements to secondees on these seven projects. A total of 14 secondees thus benefitted.

This third round of secondments offered significant opportunities for exploring alternative and more cost-effective methods of offering training placements to scientists in LatAm. The research project, coupled with secondments, offered project cohesiveness in a hybrid environment. Project PIs were able to operate more independently than at the beginning of the project, organizing their own workshops, secondments, and training, EMBL-EBI’s role shifted to one in which it supported these more decentralized operations rather than driving the agenda. This increased autonomy stimulated LatAm efforts to sustain project activities beyond the lifetime of the GCRF funding.

### 3.8 Diversity, equity, inclusion, and accessibility

DEIA is an important aspect of sound research and sound governance (Bradke et al., 2023). Research institutes in higher-income countries may have established DEIA policies and practices that can be considered, with input from partners in lower-income partner institutes, for use in their local contexts. CABANA took this approach when developing its strategy for providing access to training and workshops. We built inclusivity into all our selection processes.

CABANA’s Advisory Board included a DEIA expert who was tasked with assessing and questioning CABANA’s DEIA strategy. It is becoming customary for project funders to ensure that beneficiaries generate a DEIA strategy that embeds DEIA within its activities. Where possible, the DEIA strategy should engage with partner institutions to explore the extent to which DEIA strategy is embedded (and implemented) and to hold partner institutions accountable to it.

**Figure 8:**
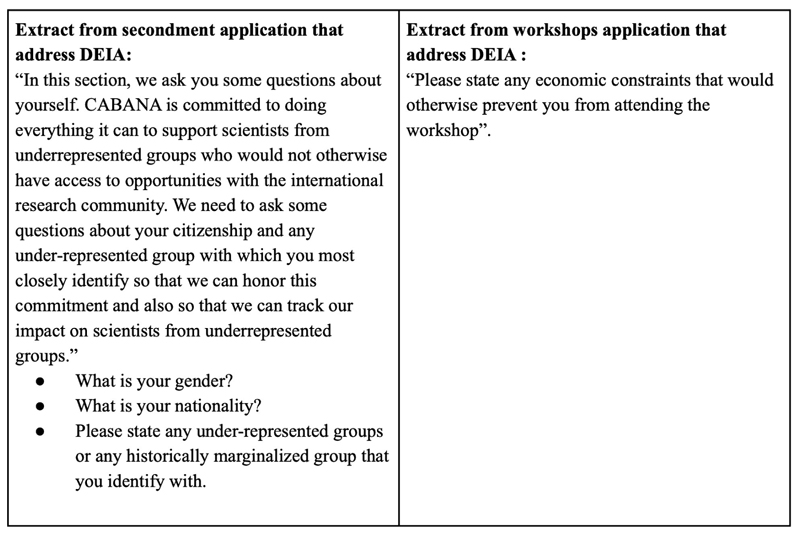
Sample of questions used to evaluate EDI to select secondees and workshop participants. These questions were part of all application forms and people were free to answer or not.

As a starting point in CABANA, all applications for attending workshops, training, and secondments included several DEIA questions (Figure 8). The selection process was collaborative and included research partners in the selection. The balance of DEIA and academic readiness was considered in the selection process.

Travel fellowships and additional support were provided in light of responses to these questions. These fellowships were awarded from 2018 to March 2020. We did not award fellowships for virtual workshops, with the exception being the joint CZI metagenomics workshop in 2020, where fellowships were awarded to all selected participants to waive registration fees. Minorities vary from country to country, and this was taken into account by our selection process.

**Figure 9:**
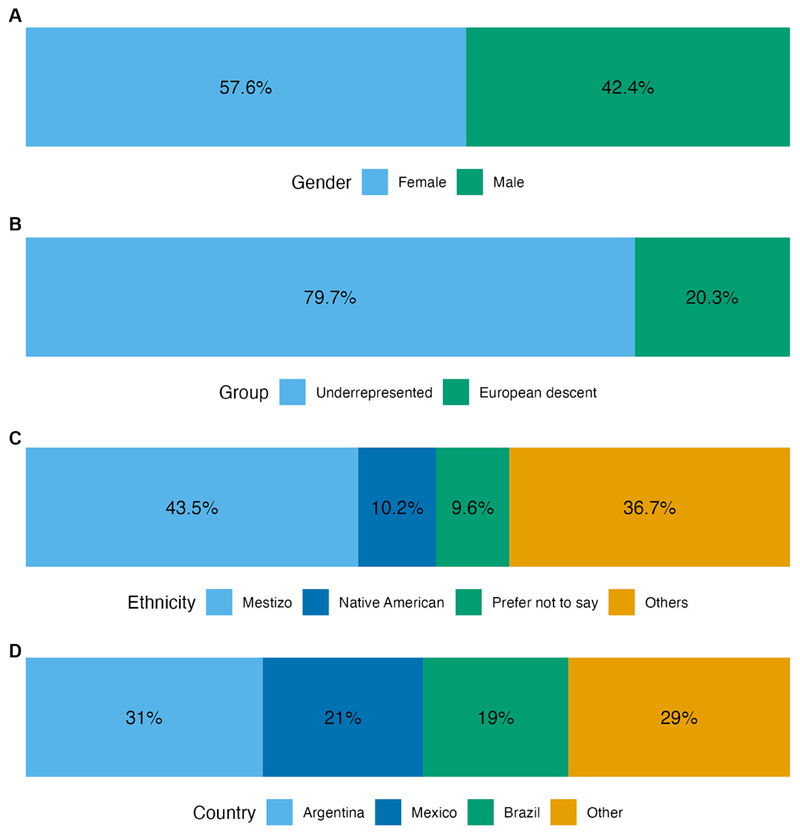
Basic statistics of fellowships and additional support awarded to attend workshops. **(A)** Gender of grantees. **(B)** Proportion of underrepresented groups. **(C)** Self-ethnicity report of grantees. **(D)** Country of origin of grantees. This data is a reflection of the EDI philosophy of the CABANA project. Female, underrepresented groups and mestizo/native American people were preferentially selected to receive financial support to attend workshops.

The summary of the workshop data provides an overview of the impact of the travel fellowships and other supportive actions on female applicants, participants from underrepresented groups including those identifying as mestizo, and Native Americans, and people from countries under economic crisis, for example, Venezuela and Argentina (Figure 9).

## 4 Discussion

From our experience of coordinating CABANA and transferring it to an expanded consortium based in LatAm, and from the evidence outlined in this paper, we believe that it is possible to develop and sustain equitable bioinformatics research and training partnerships in LatAm, focused on key global challenges of direct relevance to the UN-SDG. This has been possible because we have succeeded in increasing individual, institutional, and regional capacity. We believe that it is necessary to achieve and maintain all three for capacity to be sustained in the longer term.

In a short period, Latin American institutions have increased their capacity to generate regional research results of global importance. They have developed the capacity to generate an international training program for the region and to demonstrate how Latin American science contributes to food security, the protection of biodiversity, and the control of communicable diseases. The EMBL-EBI collaboration was key in the process, especially as host of multiple secondments and co-organizer of workshops. At the end of the project, most trainers and secondment hosts were from LatAm, providing evidence of the effectiveness of the program. We consider that EMBL-EBI is still a necessary partner in proposals led by LatAm researchers because they are an institution leading the development of global standards, data distribution, and research worldwide. They also provide connectivity with other institutions and have convening power.

Opportunities are needed to continue building capacity beyond the original six Latin American countries in CABANA. The current partners have broadened their network to include Chile, Bolivia, and El Salvador. A program like CABANA with only 10 partner institutions invested about £20,000 in each secondment to the UK, £8–10,000 in each LatAm secondment, £3–5,000 on each virtual secondment, and £15,000 in each workshop (half of the trainees were international), depending on the country. For potential LatAm and Global funders, we estimate that 10-12 secondments and 6-8 workshops per year are required to foster cohesiveness and LatAm research. This budget can be larger if more countries are involved, especially if research projects are considered to generate new data.

There is also an opportunity to develop scientific capacity beyond biodiversity, food, and health, and to encompass open science – especially open data science – more broadly. Europe was at a similar impasse in the 1970s, at the beginning of the molecular biology era. The setting up of the EMBL – Europe’s first research infrastructure for the life sciences – supported scientists in its member states (including the UK) to compete on a global scale. We urge LatAm science funders to come together to improve the research infrastructure available across the region, working multilaterally rather than through a series of independent bilateral agreements. In this way, the region could contribute hugely to science for sustainable development. Future scientific pursuits in LatAm will encounter further challenges, such as the need for increased collaboration with other developing nations in the region not included in CABANA and the integration of partners from the Caribbean.

Such an improvement in research infrastructure would complement the efforts of research charities (e.g., Wellcome Trust and CZI) and government-funded initiatives (e.g., GCRF and its successors) to support challenge-led research networks in the region and would reduce the dependency of such networks on facilities in the UK or the USA. It would support LatAm scientists to be equitably represented in global consortia for the common good, and would ultimately catalyze improved mutual trust between the global south and northern regions. Through equitable participation in organizations such as the Global Biodata Coalition (Anderson et al., 2017) and the Digital Sequence Information (DSI) Scientific Network (Scholz et al., 2022), it would be in a very strong position to contribute equitably to FAIR and open science at a volume and on a level comparable to Europe and North America thereby realizing the socioeconomic benefits of open science (Fell, 2019). We believe that sustainable development will only be possible if such progress is achieved for the region and for other underrepresented regions in the biodata landscape, including Africa and parts of the Asia–Pacific region.

Importantly, the CABANA partners and their peers must be given the opportunity as scientists to inform democracy. LatAm researchers should be supported to generate the platform for informing policymakers with the quality information they need for sound decision-making, to the level at which policy is informed in the UK and Europe. This requires a longer-term commitment to geographical reach, scientific scope, depth, equitable partnerships, and proportional scientific representation in the world’s leading research initiatives.

## Supplementary Material

Supplementary material

## Figures and Tables

**Table 1:** Description of main activities implemented by CABANA.

Name (in short)	Description	Modality
Secondments (S)	Up to six months of scientific visits to analyze data obtained or generated in the CABANA research projects or generated by independent researchers.	Virtual, in-person
Train-the-Trainer (TtT)	Workshops to teach basic theory of learning and provide tools supporting bioinformaticians to explain complex concepts and enhance their bioinformatics training skills.	Virtual, in-person
Workshops (Ws)	Cross-border bioinformatics workshops to address bioinformatics training needs in the region with local and external trainers.	Virtual, in-person
eLearning	Development of tutorials to teach essential knowledge on bioinformatics and standard data analysis pipelines. Translated to Spanish and English. Free access through cabana.online and F1000 CABANA collection.	Virtual
Research Projects (RP)	Transnational projects, selected through a lightweight application process, in which CABANA partners and new partners generate and analyze new data or analyze public data to derive new insights.	Virtual, in-person
Knowledge Exchange Meetings (KEM)	Annual meeting organized by CABANA to present the results of the activities during the year to the advisory board and other stakeholders, including funders, policymakers, and potential collaborators.	Virtual, in-person
All-hands meetings	Consortium annual meeting to celebrate achievements, assess progress, and plan for the longer term.	Virtual, in-person
Engagement meetings	Participation of partners in external meetings to share research findings and the value of bioinformatics with policymakers and other stakeholders.	In-person

**Table 2:** Downloads and views of F1000 CABANA collection tutorials up to November 2023.

Title (release year)	Downloads	Views
Analysis and exploration of microbial traits in a wet coffee fermentation experiment using MGnify (2022)	124	300
Essentials in genomics (2021)	54	294
Essentials in Metagenomics (Part I) (2020)	191	342
Essentials in Metagenomics (Part II) (2020)	482	332
Essentials Transcriptomics (2020)	88	389
Practical metagenomics: microbiome tutorial with QIIME 2 (2021)	8,341	1,471
Practical metagenomics: The Study of human gut microbiome in health and disease: Applications in Acute Diarrheal Diseases (ADD) (2021)	182	340
Practical Transcriptomics: Differential gene expression applied to food production (2021)	306	280

## Data Availability

The original contributions presented in the study are included in the article/Supplementary materials, further inquiries can be directed to the corresponding author.

## Footnotes

1
https://f1000research.com/collections/cabana

2 cabana.network/

3
www.iscb.org

4
https://wp.soibio.org/

5
https://www.metadocencia.org/

6
https://www.cabana.online/elearning

## References

[R1] Amann RI, Baichoo S, Blencowe BJ, Bork P, Borodovsky M, Brooksbank C (2019). Toward unrestricted use of public genomic data. Science.

[R2] Anderson W, Apweiler R, Bateman A, Bauer GA, Berman H, Blake JA (2017). Towards coordinated international support of Core data resources for the life sciences. bioRxiv.

[R3] Atriztán-Hernández K, Herrera-Estrella A (2022). Drosophila attack inhibits hyphal regeneration and defense mechanisms activation for the fungus *Trichoderma atroviride*. ISME J.

[R4] Ayala-Usma DA, Cárdenas M, Guyot R, Mares MCD, Bernal A, Muñoz AR (2021). A whole genome duplication drives the genome evolution of Phytophthora betacei, a closely related species to *Phytophthora infestans*. BMC Genomics.

[R5] Bradke F, Maartens A, Teichmann SA (2023). Key attributes of successful research institutes. PLOS Biol.

[R6] Bravo A, Moreno-Blanco A, Espinosa M (2023). One earth: the equilibrium between the human and the bacterial worlds †. Int J Mol Sci.

[R7] Brazas MD, Ouellette BFF (2013). Navigating the changing learning landscape: perspective from bioinformatics. Brief Bioinform.

[R8] Cabrera-Toledo D, Mendoza-Galindo E, Larranaga N, Herrera-Estrella A, Vásquez-Cruz M, Hernández-Hernández T (2022). Genomic and morphological differentiation of Spirit producing *Agave angustifolia* traditional landraces cultivated in Jalisco, Mexico. Plants.

[R9] Camarillo-Guerrero LF, Almeida A, Rangel-Pineros G, Finn RD, Lawley TD (2021). Massive expansion of human gut bacteriophage diversity. Cell.

[R10] Canto AM, Matos AHB, Godoi AB, Vieira AS, Aoyama BB, Rocha CS (2021). Multi-omics analysis suggests enhanced epileptogenesis in the Cornu Ammonis 3 of the pilocarpine model of mesial temporal lobe epilepsy. Hippocampus.

[R11] Contreras-Moreira B, Filippi CV, Naamati G, Girón CG, Allen JE, Flicek P (2021). K-mer counting and curated libraries drive efficient annotation of repeats in *plant genomes*. Plant Genome.

[R12] da Silva AL, De Abreu AP, Mariano D, Caixeta F, Santos FB, Lage FSD (2021). From in-person to the online world: insights into organizing events in bioinformatics. Front Bioinform.

[R13] Darnet S, Dragalzew AC, Amaral DB, Sousa JF, Thompson AW, Cass AN (2019). Deep evolutionary origin of limb and fin regeneration. Proc Natl Acad Sci.

[R14] Dillon MR, Bolyen E, Adamov A, Belk A, Borsom E, Burcham Z (2021). Experiences and lessons learned from two virtual, hands-on microbiome bioinformatics workshops. PLoS Comput Biol.

[R15] Fell MJ (2019). The economic impacts of open science: A rapid evidence assessment. Publications.

[R16] Filippi CV, Merino GA, Montecchia JF, Aguirre NC, Rivarola M, Naamati G (2020). Genetic diversity, population structure and linkage disequilibrium assessment among international sunflower breeding collections. Genes.

[R17] Gastauer M, Caldeira CF, Ramos SJ, Trevelin LC, Jaffé R, Oliveira G (2020). Integrating environmental variables by multivariate ordination enables the reliable estimation of mineland rehabilitation status. J Environ Manag.

[R18] Holguín AV, Cárdenas P, Prada-Peñaranda C, Leite LR, Buitrago C, Clavijo V (2019). Host resistance, genomics and population dynamics in a salmonella Enteritidis and phage system. Viruses.

[R19] Hounkpe BW, Benatti R, Carvalho BS, de Paula EV (2020). Identification of common and divergent gene expression signatures in patients with venous and arterial thrombosis using data from public repositories. PLoS One.

[R20] Hufsky F, Lamkiewicz K, Almeida A, Aouacheria A, Arighi C, Bateman A (2020). Computational strategies to combat COVID-19: useful tools to accelerate SARS-CoV-2 and coronavirus research. Brief Bioinform.

[R21] Kolympiris C, Hoenen S, Klein PG (2019). Learning by seconding: Evidence from National Science Foundation rotators. Organ Sci.

[R22] Lacarrubba-Flores MDJ, Silveira KC, Silveira C, Carvalho BS, Cavalcanti DP (2024). A mesomelic skeletal dysplasia, Kantaputra-like, not related to HOXD cluster region, and with phenotypic gender differences. Am J Méd Genet.

[R23] LatinGenomes (2020). LatinGenomes.

[R24] Laux M, Oliveira RRM, Vasconcelos S, Pires ES, Lima TGL, Pastore M (2022). New plastomes of eight Ipomoea species and four putative hybrids from eastern Amazon. PLoS One.

[R25] Lemos LN, Medeiros JD, Dini-Andreote F, Fernandes GR, Varani AM, Oliveira G (2019). Genomic signatures and co-occurrence patterns of the ultra-small Saccharimonadia (phylum CPR/Patescibacteria) suggest a symbiotic lifestyle. Mol Ecol.

[R26] Loza A, Wong-Chew RM, Jiménez-Corona M-E, Zárate S, López S, Ciria R (2023). Two-year follow-up of the COVID-19 pandemic in Mexico. Front Public Heal.

[R27] Martins A (2021). Gregorio Iraola, el científico uruguayo que mapea los microbios en los intestinos de los latinoamericanos (y por qué puede ser crucial para la salud).

[R28] Matosinho CGR, Rosse IC, Fonseca PAS, de Oliveira FS, Santos dos, Araújo FMG (2021). Identification and in silico characterization of structural and functional impacts of genetic variants in milk protein genes in the zebu breeds Guzerat and Gyr. Trop Anim Health Prod.

[R29] Maxwell L, Shreedhar P, Dauga D, McQuilton P, Terry RF, Denisiuk A (2023). FAIR, ethical, and coordinated data sharing for COVID-19 response: a scoping review and cross-sectional survey of COVID-19 data sharing platforms and registries. Lancet Digit Heal.

[R30] McGrath A, Champ K, Shang CA, van Dam E, Brooksbank C, Morgan SL (2019). From trainees to trainers to instructors: sustainably building a national capacity in bioinformatics training. PLoS Comput Biol.

[R31] Medina-Castellanos E, Villalobos-Escobedo JM, Riquelme M, Read ND, Abreu-Goodger C, Herrera-Estrella A (2018). Danger signals activate a putative innate immune system during regeneration in a filamentous fungus. PLoS Genet.

[R32] Merino GA, Saidi R, Milone DH, Stegmayer G, Martin MJ (2022). Hierarchical deep learning for predicting GO annotations by integrating protein knowledge. Bioinformatics.

[R33] Molina-Mora JA, Reales-González J, Camacho E, Duarte-Martínez F, Tsukayama P, Soto-Garita C (2023). Overview of the SARS-CoV-2 genotypes circulating in Latin America during 2021. Front Public Heal.

[R34] Moore B, Carvajal-López P, Chauke PA, Cristancho M, Angel VDD, Fernandez-Valverde SL (2021). Ten simple rules for organizing a bioinformatics training course in low- and middle-income countries. PLoS Comput Biol.

[R35] Ochoa R, Magnitov M, Laskowski RA, Cossio P, Thornton JM (2020). An automated protocol for modelling peptide substrates to proteases. BMC Bioinform.

[R36] OECD, Commission, E., America, C. D. B. of L., America, E. C. for L., Caribbean, the (2022). Latin American Economic Outlook 2022: Towards a Green and Just Transition.

[R37] Papatheodorou I, Moreno P, Manning J, Fuentes AM-P, George N, Fexova S (2019). Expression atlas update: from tissues to single cells. Nucleic Acids Res.

[R38] Payaslian F, Gradaschi V, Salazar LR, Dieterle ME, Urdániz E, Paola MD (2021a). Isolation and characterization of vB_MsmS_Celfi: a new *Mycobacterium tuberculosis* bacteriophage. PHAGE.

[R39] Payaslian F, Gradaschi V, Salazar LR, Dieterle ME, Urdániz E, Paola MD (2021b). Tightening bonds in Latin America through phage discovery. PHAGE.

[R40] Radusky L, Modenutti C, Delgado J, Bustamante JP, Vishnopolska S, Kiel C (2018). VarQ: a tool for the structural and functional analysis of human protein variants. Front Genet.

[R41] Rangel-Pineros G, Almeida A, Beracochea M, Sakharova E, Marz M, Muñoz AR (2023). VIRify: an integrated detection, annotation and taxonomic classification pipeline using virus-specific protein profile hidden Markov models. PLoS Comput Biol.

[R42] Reinholz DL, Andrews TC (2020). Change theory and theory of change: what’s the difference anyway?. Int J STEM Educ.

[R43] Rocha CS, Secolin R, Rodrigues MR, Carvalho BS, Lopes-Cendes I (2020). The Brazilian initiative on precision medicine (BIPMed): fostering genomic data-sharing of underrepresented populations. NPJ Genom Med.

[R44] Scholz AH, Freitag J, Lyal CHC, Sara R, Cepeda ML, Cancio I (2022). Multilateral benefit-sharing from digital sequence information will support both science and biodiversity conservation. Nat Commun.

[R45] Scott MF, Ladejobi O, Amer S, Bentley AR, Biernaskie J, Boden SA (2020). Multi-parent populations in crops: a toolbox integrating genomics and genetic mapping with breeding. Heredity.

[R46] Shome S, Parra RG, Fatima N, Monzon AM, Cuypers B, Moosa Y (2019). Global network of computational biology communities: ISCB’s regional student groups breaking barriers. F1000research.

[R47] Silveira KC, Kanazawa TY, Silveira C, Lacarrubba-Flores MDJ, Carvalho BS, Cavalcanti DP (2021). Molecular diagnosis in a cohort of 114 patients with rare skeletal dysplasias. Am J Méd Genet Part C.

[R48] Sriraja LO, Werhli A, Petsalaki E (2023). Phosphoproteomics data-driven signalling network inference: does it work?. Comput Struct Biotechnol J.

[R49] UK Research and Innovation (2013). Brazil partnering award: data acquisition and training for-omics in food, fuels and fisheries research.

[R50] United Nations (2015). Transforming our world: the 2030 agenda for sustainable development.

[R51] Via A, Attwood TK, Fernandes PL, Morgan SL, Schneider MV, Palagi PM (2019). A new pan-European train-the-trainer programme for bioinformatics: pilot results on feasibility, utility and sustainability of learning. Brief Bioinform.

[R52] Via A, Blicher T, Bongcam-Rudloff E, Brazas MD, Brooksbank C, Budd A (2013). Best practices in bioinformatics training for life scientists. Brief Bioinform.

[R53] Wright VA, Vaughan BW, Laurent T, Lopez R, Brooksbank C, Schneider MV (2010). Bioinformatics training: selecting an appropriate learning content management system--an example from the European bioinformatics institute. Brief Bioinform.

[R54] Yates AD, Allen J, Amode RM, Azov AG, Barba M, Becerra A (2021). Ensembl genomes 2022: an expanding genome resource for non-vertebrates. Nucleic Acids Res.

[R55] Zárate S, Taboada B, Muñoz-Medina JE, Iša P, Sanchez-Flores A, Boukadida C (2022a). The alpha variant (B.1.1.7) of SARS-CoV-2 failed to become dominant in Mexico. Microbiol Spectr.

[R56] Zárate S, Taboada B, Rosales-Rivera M, García-López R, Muñoz-Medina JE, Sanchez-Flores A (2022b). Omicron-BA.1 dispersion rates in Mexico varied according to the regional epidemic patterns and the diversity of Local Delta subvariants. Viruses.

