# Supplementary material for: The CABANA model 2017–2022: research and training synergy to facilitate bioinformatics applications in Latin America
Source: Front Educ (Lausanne). Author manuscript; Available in PMC 2024 Dec 16. (PMC7617245; doi:10.3389/feduc.2024.1358620)
Supplement: Supplementary material [file EMS200169-supplement-Supplementary_material.docx]

***Supplementary Material***

# 1. Supplementary Data 1. Logical framework for CABANA 2017-2022. *Means of Verification (MoV)* is the source of the results to the targets. It could be an annual feedback questionnaire, number of workshops, or secondee data, among others. The *Baseline* column was obtained from the baseline questionnaire at the beginning of the project. The *Target End of Project* were the objectives for the project duration. The *Achievement* contains the data of achievements at the end of the project, for most indicators we exceeded the *Target*.

| ORGANISATION NAME | EMBL EBI | | | | | | | | |
| --- | --- | --- | --- | --- | --- | --- | --- | --- | --- |
| PROJECT NAME | CABANA: Capacity Building for Bioinformatics in Latin America | | | | | | | | |
| IMPACT | Impact Indicator 1.1 | MoV | Baseline | Target End of Project | Achievement | Comments | | | Assumptions (2019) pre-Covid |
| A pan-Latin American and sustainable capacity-building program for bioinformatics research operates with at least 6 partner institutions | A network of six partner organizations organize, direct, and document a bioinformatics training program in Latin America | The baseline report and the monitoring and evaluation reports for 2019, 2020, and 2021 | 1 | 6 | 6 | At the baseline, 95% of our respondents stated that in 2017 they did not have an independently funded training program for PhD-level students and above. We have seen an encouraging and sustained upward trend of partners who state that they have an independently funded program from less than 5% in 2018 to nearly 50% in 2021.At the final evaluation, questionnaire representatives from 5 institutions (up from 4 in 2019 and 1 at the baseline) stated they have an independently funded program.At the All Hands/Hand Over meeting held in Cartagena in March 2022, the CABANA partners present were unanimous in agreeing they had at least 6 institutions providing bioinformatics training. The major change identified was the scale and scope of training now available which has increased significantly. In addition, the partners are seeking their own funding to ensure the network continues. | | | The main partners in the project continue to operate in a favorable socio and economic environment where they are able to contribute financially and with labor to the project. There is a concern that many partners anticipate an economic downturn unfavorable to their Universities' funding streams. Although some indicator numbers may appear small, each new position at a partner institution represents an additional investment to that institution of £30,000-£40,000 annually. Each new training is also a cost to that institution of approximately £5,000 |
|  | Impact Indicator 1.2 |  | Baseline | Milestone 1 October 2018 | Milestone 2 October 2019 | Milestone 3 October 2020 | Milestone 4 October 2021 | Target December 2022 |  |
|  | Number of trained and retained Bioinformaticians in target institutions above the baseline | Planned | Pending Baseline | 2 | 2 | 2 | 2 | 8 |  |
|  |  | Achieved |  |  |  |  |  |  |  |
|  |  |  | Source | | | | | |  |
|  |  |  | Review of progress and evidence from partners. This should form a part of discussions at our annual meeting as it is the project objective | | | | | |  |
|  | Impact Indicator 1.2 |  |  |  |  |  | | |  |
|  | An increase in the number of research groups incorporating new bioinformatics techniques above the baseline. (Indicator changed to: number of available positions for bioinformaticians in partner institutions) | The baseline report and the monitoring and evaluation reports for 2019, 2020, and 2021 | 4 | 6 | 21 | It was not feasible to track the new bioinformatics techniques used by our partners because there was already a broad spectrum of techniques used. The project identified "the number of vacancies" as a noteworthy indicator to track the demand for bioinformatics and the availability of funding. At the baseline report the CABANA partners declared they had 4 Bioinformatics vacancies in 2018. The project had 8 vacancies in 2019 and 2020, we had 5 vacancies in 2021. This is an increase of 21 vacancies over the project period. These are new vacancies because output indicator 1.1 below notes an increase of 25 recruited and retained new bioinformatics positions. Partners also reported a total of 10-15 positions being opened every year. We defined a bioinformatics position as a scientist spending 30% of their time on bioinformatics rather than wet lab work. | | |  |
|  | Impact Indicator 1. 4 |  |  |  |  |  | | |  |
|  | Number of independently funded short courses conducted (above 2017 levels in partner and new institutions) | The baseline report and the monitoring and evaluation reports for 2019, 2020, and 2021 | 31 | 6 | Year 2019, 43 courses, an increase of 12 from the baseline | In 2019, the CABANA partners held 12 independently funded short courses, which was double the project target. This level of activity was not sustained in 2020 and 2021. The main explanations can be that partners focussed on achievable CABANA activities where funding was available in the context of the Covid-19 pandemic where many University campuses were closed and funding resources and local logistics support seriously constrained. | | |  |
|  |  |  |  |  | Year 2020, 22 courses, a decrease of 9 from the baseline |  |  |  |  |
|  |  |  |  |  | Year 2021, 18 courses, a decrease of 13 from the baseline |  |  |  |  |
| OUTCOME1 | Outcome Indicator 1.1 |  |  |  |  |  | | |  |
|  | Impact Indicator 1.2 |  | Baseline | Target December 2022 | Milestone 2 October 2019 |  |  |  |  |
| Partner institutions (have developed a critical mass of bioinformaticians and scientists able to) conduct collaborative Pan-Latin American bioinformatics research at the highest international level in infectious diseases, food security, and biodiversity [to discuss with partners what critical mass looks like in their institutions]. | Number of trained and retained Bioinformaticians in target institutions above the baseline. | The baseline report and the monitoring and evaluation reports for 2019, 2020, 2021 | 30 | 38 | 55 | We note that the number of Bioinformaticians has been sustained through the pandemic.The number of bioinformatics positions has risen from approximately 30 at the start of the project to 55. Some institutions have seen a drop since last year, whereas others have managed to sustain or even increase the number of positions. This variance is perhaps not surprising given the challenging global economy. | | |  |
|  | Outcome Indicator 1.2 |  |  |  |  |  | | |  |
|  | Number of collaborative research projects involving partners and other Latin American projects above the baseline. | The baseline report and the monitoring and evaluation report for 2019, 2020 and 2021 | *9* | *8* | *16* | There was an increased level of research collaborations at the regional level. The baseline anticipated 8 additional projects above the baseline. By the end of the project, partners had secured another 15 projects in the Latin American region. There were on average 5 extra regional projects above the baseline each year. The national level of collaboration among partners increased from a baseline of 34 in 2018 to 44 in 2020. This number dropped below the baseline level in 2022 to 26 projects. But in total, we have seen an increase in collaborations. | | |  |
|  |  |  |  |  |  |  |  |  |  |
|  | Outcome Indicator 1.3 |  |  |  |  |  | | |  |
|  | Improved balance/distribution of bioinformatics (publications) between partners. |  | N/A | 40% | 85% | 95% of partners reported submitting their data to publicly available databases. More than half of the CABANA partners believed that 85% of their colleagues also made their data publicly available. We have clear evidence from CABANA secondees that sharing data is the norm. Our original baseline data was inconclusive because some of the partners responded in numbers of colleagues and others responded in percentage of colleagues. The question was reworded in subsequent years. | | |  |
| OUTCOME 2 | Outcome Indicator 2.1 |  |  |  |  |  | | | Assumptions |
| 30% increase above the baseline of reported national and international meetings to discuss research challenges and research opportunities in L.American region. | Number of policy meetings above the baseline raising the importance of bioinformatics research in the three project themes, nationally. | The baseline report and the monitoring and evaluation reports for 2019, 2020, and 2021 | 31 | 37 | 43 | The CABANA project identified early that more engagement with policymakers in Latin America was necessary to create sustainability in the training program. The aim is for our partners to become key informants by sharing research findings and the value of bioinformatics with policymakers and stakeholders. Our partners have demonstrated an increase in engagement with key policymakers at meetings. Our conference programme has been restricted in 2020-2021, but four live conferences are planned in 2022. | | |  |
|  | Outcome Indicator 2.2 |  |  |  |  |  | | |  |
|  | Number of policy meetings above the baseline raising the importance of bioinformatics resources for informed decision making. in the three project themes. Regionaly. | The baseline report and the monitoring and evaluation reports for 2019, 2020, and 2021 | 23 | 27 | 58 | On average 10 meetings per year above the baseline of 23 meetings were held each year 39 meetings were held in 2019, 34 meetings were held in 2020, and 27 meetings in 2021. We note a decrease in policy meetings towards the end of the project, which is probably linked to the Covid-19 pandemic. | | |  |
|  | Outcome Indicator 2.3 |  |  |  |  |  | | |  |
|  | Number of new researchers making their data available to international data centers. | 3 secondee questionnaires and the baseline report, plus the monitoring and evaluation reports for 2019, 2020, and 2021 | 0 | 21 | 24 | We were unable to track the number of "new researchers", but we have tracked the performance of our secondees who came to the UK and those who remained studying in Latin America. Of the 39 secondees 28 stated they had uploaded material onto a publicly available database or were comfortable they would do so soon. There is also a general acceptance of making bioinformatics data available on public access databases 60% of our partners stated that 85% or more of their colleagues made their data available on publicly available databases. We also have 28 publications that will have made their data public and all the elearning courses are using Latin American data as part of the training exercises. | | |  |
|  | Outcome Indicator 2.4 |  |  |  |  |  | | |  |
|  | Number of available positions for bioinformaticians in partner institutions above the baseline. | The baseline report and the monitoring and evaluation reports for 2019, 2020, and 2021 | 4 | 8 | 21 | There was a total of 4 vacant bioinformatics vacancies at the baseline, there were a total of 21 vacancies over the following 3 years, an average of 7 per year. In addition a further 55 bioinformaticians were recruited by the CABANA partners. | | |  |
|  | Outcome Indicator 2.5 |  |  |  |  |  | | |  |
|  | The number of collaborative research projects involving partners and international institutions above the baseline. | The baseline report and the monitoring and evaluation reports for 2019, 2020, and 2021 | 4 | 7 | 21 | There has been a significant increase in the number of International collaborations at our partner institutions. Our target was 7 collaborations, the partners have been involved in 21. | | |  |
| OUTPUT 1 | Output Indicator 1.1 |  |  |  |  |  | | | Assumption |
| Research Secondments Implemented. | Number of Secondments. | Activity data. Records of the number of secondees who participated in the project | 0 | 28 | 39 | The key output indicator of secondments has been exceeded. 20 live/in situ secondments have been completed in the UK, 11 in the first round and 9 in the second round, of which:19 at EMBL-EBI1 The Earlham InstituteTwo have been completed in Latin America:1 at Universidad de San Martín de Porres, Lima, Peru with Professor Ricardo Fujita1 at The University of Buenos Aires with Professor TurjanskiDuring the COVID-19 travel restrictions, we promoted a more regional and virtual approach to secondments.16 secondments were placed in Latin America in virtual settings, 13 were associated with the Research Innovation Awards, two were based in Venezuela, and one virtually with Public Health England.The provenance of our secondees is as follows: 4 Brazil, 11 Argentina, 5 Mexico, 9 Colombia, 3 Costa Rica, 4 Peru, Venezuela 2. 50% are female. | | | Output 1.2 difficult to document outcomes beyond the lifetime of the project. Output 1.3 allowing one year after the secondment for publication to materialize |
|  | Output Indicator 1.2 |  |  |  |  |  | | |  |
|  | Active collaborations between host and secondee laboratory after 2 years. | Records of the number of secondees who participated in the project and anecdotal evidence. | 0 | 8 | 8 | We are unclear about the number of partners actively involved in research collaborations with secondees. Of the first two secondees cohorts alone, we know that we have 8 ongoing relationships with their host research group. We have not delved into greater detail, but the relationship is likely to be over information sharing and uploading sequencing information. We also know of two CABANA partners that have an ongoing collaborative relationship with EMBL-EBI, one at the University of Buenos Aires and the other at San Martin de Porres. The project representative of the VALE Institute has provided advice on tracking biodiversity and conservation to EMBL-EBI. | | |  |
|  | Output Indicator 1.3 |  |  |  |  |  | | |  |
|  | Number of joint papers and data uploaded into publicly available databases as a direct consequence of secondments | Project tracking and sharing of publication information | 0 | 21 | 28 21 Publications and 7DOIs *needs Updating* | All the publications are attributable to the CABANA project. The project and GROW UKRI are given due recognition. We anticipate more publications in the next year. | | |  |
| OUTPUT 2 | Output Indicator 2.1 |  |  |  |  |  | | | Assumption |
| Train The Trainer Workshops Conducted. | Number of trainers developed. | Tracking of project activities | 0 | 36 | 123 | The key indicator of Output 2 has been exceeded. A total of 4 workshops associated with Bioinformatics courses have been delivered a further 9 additional virtual courses have been delivered. The project has supported 123 new trainers some are associated with the CABANA partner institutions others are from other institutions in Latin America. | | |  |
|  | Output Indicator 2.2 |  |  |  |  |  | | |  |
|  | Number of courses delivered by all trainers | Tracking of project activities |  | 62 | 33 new trainers have participated in a total of 36 workshops. Seven Elearning courses have been devised by one of the trainnees. | We have 33 new trainers who have participated in training courses. Many of these have participated in more than one training workshop, and indeed one of the trained trainers has delivered 7 e-learning training courses for the project. In addition 19 training sessions were run by trainers as part of the UniAndes MSc EBI module. | | |  |
|  | Output Indicator 2.3 |  |  |  |  |  | | |  |
|  | Number of new trainees per partner institution trained. |  | 0 | 124 | 35 | 35 trainers are members of our partner institutions. With the benefit of hindsight, having more trainers distributed outside of our partner institutions is more beneficial. | | |  |
|  | Output Indicator 2.4 |  |  |  |  |  | | |  |
|  | Number of new trainers in all partner institutions retained, or career path further secured for 2+ years. |  | 0 | 25 | 25 | We have not been able to track all the trained trainers. We are encouraged by Outcome Indicator 1.1, where 25 new bioinformatics positions have been created during the project period in a challenging funding environment. | | |  |
| OUTPUT 3 | Output Indicator 3.1 |  |  |  |  |  | | | Assumption |
| Short Courses designed and implemented. | Number of short courses conducted. | Tracking of project activities | 0 | 28 | 28 | The indicator of Output 3 has been met. The project delivered 28 Bioinformatics courses. In the initial stage of the project, the courses were "in situ". During the Covid pandemic and ensuing lockdown, we moved the training program to virtual courses. The course content remained rigorous, the courses were spread over several days and included coursework to be conducted by the attendees. The participants' feedback forms continue to show high satisfaction with the course content and learning. | | |  |
|  | Output Indicator 3.2 |  |  |  |  |  | | |  |
|  | Number of individuals trained. | Tracking of project activities | 0 | 600 | 835 | A total of 835 students participated in the training courses. Our early "in situ" courses had room for 20 attendees. Once we were obliged to move to a virtual setting, we were able to accept more attendees and had a target of 30-40 attendees per workshop. We maintained our equality, diversity, and inclusion standards. A mix of virtual, hybrid, and in situ workshops reduces the cost of conducting workshops and offers opportunities to create a sustainable training program for the whole of Latin America. | | |  |
|  | Output Indicator 3.3 |  |  |  |  |  | | |  |
|  | Quality Control feedback loop on Trainers. | Monitoring and evaluation forms of attendees on each course | 0 | 28 | 28 | The CABANA project has a rigorous monitoring system in place that follows the career paths of secondees who have become trainers (3 monitoring questionnaires) and monitors the level of satisfaction of trainees at the training. The feedback from workshops has highlighted a high degree of satisfaction with the course with 80% satisfied or highly satisfied. In addition, CABANA partner PIs have maintained a working relationship with many of the trainers and provided teaching opportunities when available. | | |  |
|  |  | Evaluation by course organizer at EMBL-EBI |  |  |  |  |  |  |  |
|  |  | Mentoring by Latin American Course organizer |  |  |  |  |  |  |  |
| OUTPUT 4 | Output Indicator 4.1 |  |  |  |  |  | | | Assumptions |
| E-learning resources created and used. | Number of courses with Latin American context and input incorporated in the course. | Training material created and made available on CABANA websites. | 0 | 9 | 9+1 extra short course and 2 in the pipeline. | The key indicator of output 4 has been exceeded. The 9 courses in Spanish and English have been created under the leadership of the University of Costa Rica and UniAndes Colombia. The training material is accessible on the CABANA (EBI) and the CABANA LatAm websites, managed by the University of Buenos Aires. Two additional courses are being finalized by the University de Los Andes in Colombia. In addition, course material has been created to introduce Undergraduates to Bioinformatics. The creation of many of the training courses has been led by CABANA secondees. | | |  |
|  | Output Indicator 4.2 |  |  |  |  |  | | |  |
|  | Number of courses incorporated into L America Curriculum. | Anecdotal evidence | 0 | 4 | 4 | UniAndes, University of Costa Rica, University of Buenos Aires, and Cinvestav have all declared their interest in using the courses as part of their curriculum | | |  |
|  | Output Indicator 4.3 |  |  |  |  |  | | |  |
|  | The number of additional training activities that CABANA has contributed. (short courses etc.). | Tracking of project activities | 0 | 6 | 12 | The CABANA project conducted 9 additional train-the-trainer courses we also conducted two courses entitled "Bioinformatics for PIs". The CABANA partners also organized an International symposium/knowledge exchange on SARS-CoV-2 in 2020. | | |  |
|  | Output Indicator 4.4 |  |  |  |  |  | | |  |
|  | E-learning infrastructure in each partner. | Accepted Innovation Award infrastructure call for proposals | 0 | 3 | 5 | 5 Partner institutions have benefitted from improved infrastructure in the project. INTA Argentina requested additional infrastructure as part of the proposal submission. A further 4 partners, the University of Buenos Aires, the University of Costa Rica, the University de Los Andes, and Langebio/Cinvestav Mexico, requested improved e-learning training infrastructure as part of the project's infrastructure innovation award, which made awards of up to £8,000 to improve training facilities and support virtual training using a "hub and spoke" structure at the national and regional level. | | |  |
|  | Output Indicator 4.5 |  |  |  |  |  | | |  |
|  | Number of course participants from Latin America above 2017 levels. |  | 0 | 135 | 290 | The number of participants from Latin America has significantly increased, many of the participants have also become trainers and participated on the CABANA project as secondees. | | |  |
| OUTPUT 5 | Output Indicator 5.1 |  |  |  |  |  | | | Assumptions |
| 70% Partners participate in internal calls for proposals for collaborative research and Elearning infrastructure support to enhance training. | 5 Partners participate in Innovation call for proposals. | Research Calls for proposals completed | 0 | 5 | 8 | All Partners have participated in innovation award research collaborations, many of them on multiple projects. Two institutions, CINVESTAV and The VALE Institute have demonstrated leadership in sequencing capacity. UBA Argentina and CINVESTAV were involved in 4 projects. | | |  |
|  | Output Indicator 5.3 |  |  |  |  |  | | |  |
|  | A training database is developed and managed by Latin American institutions. | Database created | 0 | 1 | 1 | The trainer database forms part of the CABANA website, which UBA manages in Latin America. One of our secondees, Maria Bernadi, has led developing the trainer database. The CABANA partnership is currently seeking additional funding to increase the scope of the website. The nine Elearning courses are also on the database. | | |  |

# 2. Supplementary Data 2. Baseline questionnaire, and Monitoring and Evaluation questionnaire. This questionnaire was used at the beginning of the project to set the baseline. Then it was completed every year by the CABANA partners to monitor and evaluate progress. This is an example that evaluated the period July 2019 to July 2020

1. Name.

Answer: text

2. Institution.

Answer: text

3. Do you currently have an independently funded training program for Bioinformaticians at the MSc level and above at your institution?

Answers: No, Yes

4. Between July 2019 and July 2020, how many Bioinformaticians for MSc level and above did your institution train using independent funding? Please provide a number.*Bioinformatician = staff performing Bioinformatics analysis at least 30% of their time that could lead to a publication or uploading of data to public repositories.

Answer: text

5. Between July 2019 and July 2020, in your own faculty, how many Bioinformaticians* are funded? *Bioinformatician = staff performing Bioinformatics analysis at least 30% of their time that could lead to a publication or uploading of data to public repositories.

Answer: A number from 0 to 10 or more

6. Between July 2019 and July 2020, in your own faculty, how many Bioinformatician posts have been opened? *Bioinformatician = staff performing Bioinformatics analysis at least 30% of their time that could lead to a publication or uploading of data to public repositories.

Answer: A number from 0 to 10 or more

7. Between July 2019 and July 2020, in your own faculty, how many Bioinformatician posts remain vacant (as of July 2020)? *Bioinformatician = staff performing Bioinformatics analysis at least 30% of their time that could lead to a publication or uploading of data to public repositories.

Answer: A number from 0 to 10 or more

8. Between July 2019 and July 2020, how many of Bioinformaticians* did you work with directly on projects? *Bioinformatician = staff performing Bioinformatics analysis at least 30% of their time that could lead to a publication or uploading of data to public repositories.

Answer: A number from 0 to 10 or more

9. How many co-funded with CABANA short courses in Bioinformatics research for PhD level and above have you hosted or co-hosted outside of your institution between July 2019 and July 2020.

Answer: A number from 0 to 10 or more

10. How many independently funded outside of CABANA short courses in Bioinformatics research for PhD level and above have you hosted or co-hosted outside of your institution between July 2019 and July 2020.

Answer: A number from 0 to 10 or more

11. Between July 2019 and July 2020, what percentage of the hosted courses (see above) had studies or worked examples in a Latin American context? Please provide a number.

Answer: A number

12. Between July 2019 and July 2020 for how many national collaborative research projects involving bioinformatics were you a funded investigator?

Answer: A number from 0 to 10 or more

13. Between July 2019 and July 2020, how many transnational projects (with other Latin American countries) involving bioinformatics were you a funded investigator?

Answer: A number from 0 to 10 or more

14. Between July 2019 and July 2020, for how many collaborative research projects with international (non Latin American) partners involving bioinformatics were you a funded investigator?

Answer: A number from 0 to 10 or more

15. Between July 2019 and July 2020, how many projects for which you are an investigator have enabled the leveraging of new bioinformatics projects?

Answer: A number from 0 to 10 or more

16. Between July 2019 and July 2020, how many workshops did you or a representative of your department (or institution) showcase your bioinformatics research in your country?

Answer: A number from 0 to 10 or more

17. Between July 2019 and July 2020, how many workshops did you or a representative of your department (or institution) showcase your bioinformatics research in Latin America?

Answer: A number from 0 to 10 or more

18. Between July 2019 and July 2020, how many workshops did you or a representative of your department (or institution) showcase your bioinformatics research outside of Latin America?

Answer: A number from 0 to 10 or more

19. Between July 2019 and July 2020, how many external meetings did you or a representative of your department (or institution) attend to discuss the challenges of bioinformatics resources or capacity in your country?

Answer: A number from 0 to 10 or more

20. How many of your close work colleagues who routinely use bioinformatics and sequencing in their research routinely submit their data to international open-access bioinformatics databases

1. Up to 20% of my close work colleagues
2. Up to 40% of my close work colleagues
3. Up to 60% of my close work colleagues
4. Up to 85% of my close work colleagues
5. 100% of my close work colleagues
6. Not sure

21. Do you as a co-PI on the CABANA project submit your data to international open-access bioinformatics databases?

Answers: Yes, No

22. Between July 2019 and July 2020, how many secondments (other than CABANA) of more than 3 months were your immediate colleagues, at PhD and above, able to attend?

Answer: A number from 0 to 10 or more

23. Between July 2019 and July 2020, for your institution, how many new bioinformatics Trainers became operational?

Answer: A number from 0 to 10 or more, or not sure

24. Between July 2019 and July 2020, how many courses did each Trainer deliver or contribute to?

Answer: A number from 0 to 10 or more, or not sure

25. Between July 2019 and July 2020, how many new trainees at PhD level and above benefitted from a new trainer? Please provide a number.

Answer: A number

26. How effective has CABANA been so far at helping to build a pan-Latin American, sustainable capacity-building program for bioinformatics research? Please tick the boxes if you agree to the statements below. Since CABANA started:

1. The bioinformatics research at my Institute really took off
2. The number of our pan-Latin American collaborations has increased noticeably
3. We have submitted one or more papers with Latin American co-authors only
4. My hope for a long-term career in Bioinformatics in Latin America has grown
5. To my knowledge, the number of independently funded Latin American workshops has increased
6. The general quality of Bioinformatics candidates at our institution has increased
7. Limited impact on the quality/quantity of the bioinformatics research from my institute has been observed
8. Not aware of any independently funded workshops being organized in Latin America
9. Many of the researchers that are trained are choosing to continue their research in Europe or the USA
10. Any effect of the CABANA project is likely to be dwarfed by the very limited funding across LatAm

26. Between July 2019 and July 2020, has your involvement and participation in bioinformatics meetings in your faculty changed? (tick up to 2):

1. My involvement has Increased
2. I have more information to advise
3. I have less information to advise
4. My involvement has decreased

27. Between July 2019 and July 2020, has your involvement and participation changed in budget allocation meetings where you could argue the case for investment in bioinformatics? (tick up to 2):

1. My involvement has Increased
2. I have more information to advise colleagues
3. My Involvement has remained the same
4. I have less information to advise colleagues
5. My involvement has decreased

28. Between July 2019 and July 2020, has your involvement and participation in conferences regarding bioinformatics changed? (tick up to 2):

1. My involvement has Increased
2. I have more information to advise
3. My Involvement has remained the same
4. I have less information to advise
5. My involvement has decreased

29. Between July 2019 and July 2020, has your involvement and participation changed in meetings with politicians and key influencers to promote bioinformatics research in your region? (tick up to 2):

1. My involvement has Increased
2. I have more information to advise
3. My Involvement has remained the same
4. I have less information to advise
5. My involvement has decreased

30. Between July 2019 and July 2020, has the level of opportunities for funding bioinformatics activities in your region (including the work of CABANA) changed? (tick up to 2):

1. National and regional opportunities have increased
2. International opportunities have increased
3. On average, opportunities have remained the same
4. National and regional opportunities have decreased
5. International opportunities have decreased

31. Between July 2019 and July 2020, did you experience any barriers to bioinformatics research in 2018? If so, what were they?

Answer: Text

# 3. Supplementary Data 3. Basic data of publications attributed to the CABANA project from 2017 to 2023, including type and theme they are associated with, shared resources, and organism of study where it applies. Not applicable (NA).

| **DOI** | **Title** | **Type** | **Theme** | **Year of publication** | **Data or script location** | **Organism of study/ Environment studied** | **Other products** |
| --- | --- | --- | --- | --- | --- | --- | --- |
| <https://doi.org/10.7490/f1000research.1118734.1> | Practical metagenomics: microbiome tutorial with QIIME 2 | eLearning tutorial | Biodiversity | 2021 | NA | Lithobates vibicarius | NA |
| <https://doi.org/10.7490/f1000research.1118938.1> | Analysis and exploration of microbial traits in a wet coffee fermentation experiment using MGnify | eLearning tutorial | Food security | 2022 | NA | Ecuadorian coffee | NA |
| <https://f1000research.com/documents/10-307> | Practical Transcriptomics: Differential gene expression applied to food production | eLearning tutorial | Food security | 2021 | NA | Coffee | NA |
| <https://doi.org/10.7490/f1000research.1118763.1> | Practical metagenomics: The Study of human gut microbiome in health and disease: Applications in Acute Diarrheal Diseases (ADD) | eLearning tutorial | Communicable diseases | 2021 | NA | Human gut | NA |
| <https://doi.org/10.7490/f1000research.1118491.1> | Essentials in Genomics | eLearning tutorial | Other | 2021 | NA | NA | NA |
| <https://doi.org/10.7490/f1000research.1118333.1> | Essentials in Metagenomics (Part I) | eLearning tutorial | Other | 2020 | NA | NA | NA |
| <https://doi.org/10.7490/f1000research.1118334.1> | Essentials in Metagenomics (Part II) | eLearning tutorial | Other | 2020 | NA | NA | NA |
| <https://doi.org/10.7490/f1000research.1117945.1> | Essentials Transcriptomics | eLearning tutorial | Other | 2020 | NA | NA | NA |
| <https://doi.org/10.1371/journal.pcbi.1009056> | Experiences and lessons learned from two virtual, hands-on microbiome bioinformatics workshops | Research article | Biodiversity | 2021 | NA | NA | NA |
| <https://doi.org/10.1111/mec.15208> | Genomic signatures and co-occurrence patterns of the ultra-small Saccharimonadia (phylum CPR/Patescibacteria) suggest a symbiotic lifestyle | Research article | Biodiversity | 2019 | NA | Saccharimonadia | NA |
| <https://doi.org/10.1016/j.jenvman.2019.109894> | Integrating environmental variables by multivariate ordination enables the reliable estimation of mineland rehabilitation status | Research article | Biodiversity | 2020 | NA | Mineland | NA |
| <https://doi.org/10.1002/tpg2.20143> | K-mer counting and curated libraries drive efficient annotation of repeats in plant genomes | Research article | Biodiversity | 2021 | <https://github.com/Ensembl/plant-scripts> | Plant genomes | NA |
| <https://doi.org/10.1002/edn3.34> | Vertebrate diversity revealed by metabarcoding of bulk arthropod samples from tropical forests | Research article | Biodiversity | 2019 | <https://erda.ku.dk/public/archives/11f676c75a8fee6e34c5e8034bfad3c9/published-archive.html> | Arthropods | NA |
| <https://doi.org/10.1186/s12864-021-08079-y> | A whole genome duplication drives the genome evolution of Phytophthora betacei, a closely related species to Phytophthora infestans | Research article | Food security | 2021 | PRJNA608953 for P. betacei P8084 and PRJNA517953 for P. infestans RC1-10 | Phytophthora betacei | NA |
| <https://doi.org/10.3389/fsoil.2022.835849> | Agriculture by Irrigation Modifies Microbial Communities and Soil Functions Associated With Enhancing C Uptake of a Steppe Semi-Arid Soil in Northern Patagonia | Research article | Food security | 2022 | SAMN23482046 | Soil | NA |
| <https://doi.org/10.1371/journal.pgen.1007390> | Danger signals activate a putative innate immune system during regeneration in a filamentous fungus | Research article | Food security | 2022 | GSE115811 | Trichoderma atroviride | NA |
| <https://doi.org/10.1038/s41396-021-01068-9> | Drosophila attack inhibits hyphal regeneration and defense mechanisms activation for the fungus Trichoderma atroviride | Research article | Food security | 2022 | NA | Trichoderma atroviride | NA |
| <https://doi.org/10.3390/genes11030283> | Genetic Diversity, Population Structure and Linkage Disequilibrium Assessment among International Sunflower Breeding Collections | Research article | Food security | 2020 | NA | Helianthus annuus spp. macrocarpus | NA |
| <https://doi.org/10.1089/phage.2020.0029> | Genome Sequence and Characterization of Lactobacillus casei Phage, vB_LcaM_Lbab1 Isolated from Raw Milk | Research article | Food security | 2021 | NA | Lactobacillus casei | NA |
| <https://doi.org/10.3390/plants11172274> | Genomic and Morphological Differentiation of Spirit Producing Agave angustifolia Traditional Landraces Cultivated in Jalisco, Mexico | Research article | Food security | 2022 | Request data from authors | Agave angustifolia | NA |
| <https://doi.org/10.3390/v14061165> | Dominance of Three Sublineages of the SARS-CoV-2 Delta Variant in Mexico | Research article | Communicable diseases | 2022 | GISAID repository and CoViGen genomes | SARS-CoV-2 | NA |
| <https://doi.org/10.1101/2021.03.23.436718> | Exploring Leishmania-Host Interaction with Reactome, a Database of Biological Pathways and Processes | Research article | Communicable diseases | 2021 | NA | Leishmania | NA |
| <https://doi.org/10.3390/v11020188> | Host Resistance, Genomics and Population Dynamics in a Salmonella Enteritidis and Phage System | Research article | Communicable diseases | 2019 | NA | Salmonella enteritidis | NA |
| <https://doi.org/10.1089/phage.2020.0030> | Isolation and Characterization of vB_MsmS_Celfi: A New Mycobacterium tuberculosis Bacteriophage | Research article | Communicable diseases | 2021 | NA | Mycobacterium tuberculosis | NA |
| <https://doi.org/10.1016/j.cell.2021.01.029> | Massive expansion of human gut bacteriophage diversity | Research article | Communicable diseases | 2021 | Public data from ENA | Bacteriophages | NA |
| <https://doi.org/10.3390/v15010243> | Omicron-BA.1 Dispersion Rates in Mexico Varied According to the Regional Epidemic Patterns and the Diversity of Local Delta Subvariants | Research article | Communicable diseases | 2023 | EPI_SET_220927gw | SARS-CoV-2 | NA |
| <https://doi.org/10.3389/fpubh.2023.1095202> | Overview of the SARS-CoV-2 genotypes circulating in Latin America during 2021 | Research article | Communicable diseases | 2023 | Public data | SARS-CoV-2 | NA |
| <https://doi.org/10.1007/s15010-023-02034-7> | SARS-CoV-2 BW.1, a fast-growing Omicron variant from southeast Mexico bearing relevant escape mutations | Research article | Communicable diseases | 2023 | EPI_SET_230222ws, https://github.com/GAL-Repository/SARS-CoV-2_BW_lineage | SARS-CoV-2 | NA |
| <https://doi.org/10.1128/spectrum.02240-21> | The Alpha Variant (B.1.1.7) of SARS-CoV-2 Failed to Become Dominant in Mexico | Research article | Communicable diseases | 2022 | GISAID and GenBank | SARS-CoV-2 | NA |
| <https://doi.org/10.3389/fpubh.2022.1050673> | Two-year follow-up of the COVID-19 pandemic in Mexico | Research article | Communicable diseases | 2023 | Request data to authors | SARS-CoV-2 | NA |
| <https://doi.org/10.1371/journal.pcbi.1011422> | VIRify: An integrated detection, annotation and taxonomic classification pipeline using virus-specific protein profile hidden Markov models | Research article | Communicable diseases | 2023 | <https://github.com/EBI-Metagenomics/emg-viral-pipeline> | Viruses | NA |
| <https://doi.org/10.1101/098996> | A global perspective on bioinformatics training needs | Research article | Other | 2017 | NA | NA | NA |
| <https://doi.org/10.1186/s12859-020-03931-6> | An automated protocol for modeling peptide substrates to proteases | Research article | Other | 2020 | https://github.com/rochoa85/Modelling-Protease-Substrates, https://github.com/magnitov/protease_annotation_pipeline | NA | NA |
| <https://doi.org/10.15517/rbt.v69i4.46873> | Bioinformatics advances in Costa Rica: retrospective view and perspectives | Research article | Other | 2021 | NA | NA | NA |
| <https://doi.org/10.3389/feduc.2021.710971> | Challenges and Considerations for Delivering Bioinformatics Training in LMICs: Perspectives From Pan-African and Latin American Bioinformatics Networks | Research article | Other | 2021 | NA | NA | NA |
| <https://doi.org/10.15381/rpb.v28i1.17867> | Data mining of DNA sequences submitted by Peruvian institutions to public genetic databases | Research article | Other | 2021 | NA | NA | NA |
| <https://doi.org/10.1073/pnas.1900475116> | Deep evolutionary origin of limb and fin regeneration | Research article | Other | 2019 | [PRJNA480693](http://www.ncbi.nlm.nih.gov/sra/?term=PRJNA480693) and [PRJNA480698](http://www.ncbi.nlm.nih.gov/sra/?term=PRJNA480698) | Sarcopterygians | NA |
| <https://doi.org/10.1093/nar/gkab1007> | Ensembl Genomes 2022: an expanding genome resource for non-vertebrates | Research article | Other | 2022 | <https://github.com/Ensembl/ensembl-production-imported> | Non-vertebrates | Genome resource |
| <https://doi.org/10.1371/journal.pone.0235501> | Identification of common and divergent gene expression signatures in patients with venous and arterial thrombosis using data from public repositories | Research article | Other | 2020 | Public repositories | Homo sapiens | NA |
| <https://doi.org/10.1016/j.cpc.2020.107716> | PARCE: Protocol for Amino Acid Refinement through Computational Evolution | Research article | Other | 2021 | <https://github.com/PARCE-project/PARCE-1> | NA | Bionformatics tool |
| <https://doi.org/10.1016/j.csbj.2022.12.010> | Phosphoproteomics data-driven signaling network inference: does it work? | Research article | Other | 2023 | NA | NA | NA |
| <https://doi.org/10.1371/journal.pcbi.1010220> | Ten simple rules for leveraging virtual interaction to build higher-level learning into bioinformatics short courses | Research article | Other | 2022 | NA | NA | NA |
| <https://doi.org/10.1371/journal.pcbi.1009218> | Ten simple rules for organizing a bioinformatics training course in low- and middle-income countries | Research article | Other | 2021 | NA | NA | NA |
| <https://doi.org/10.1038/s41525-020-00149-6> | The Brazilian Initiative on Precision Medicine (BIPMed): fostering genomic data-sharing of underrepresented populations | Research article | Other | 2020 | https://github.com/crirocha/BIPMed/ and https://github.com/labbcb/bipmed-analysis/blob/master/BIPMed_analysis.md, PRJEB39251, GSE156652, ERZ1463065 | NA | NA |
| <https://doi.org/10.1093/nar/gkab1127> | The European Bioinformatics Institute (EMBL-EBI) in 2021 | Research article | Other | 2021 | NA | NA | NA |
| <https://doi.org/10.1089/phage.2020.0028> | Tightening Bonds in Latin America Through Phage Discovery | Research article | Other | 2021 | NA | NA | NA |
| <https://doi.org/10.3389/fgene.2018.00620> | VarQ: A Tool for the Structural and Functional Analysis of Human Protein Variants. | Research article | Other | 2018 | NA | NA | Bionformatics tool |

#

# 4. Supplementary Data 4. Summary of Train the Trainer workshops of the CABANA project

| **Title** | **Institution** | **Location** | **Country** | **Date** | **Hosts** | **Format** | **CABANA or supported?** |
| --- | --- | --- | --- | --- | --- | --- | --- |
| Train the Trainer Honduras |  | Virtual | Honduras | 22-23 June 2020 | Patricia Carvajal-López, Maria Trinidad Bernardi | Virtual | CABANA |
| Train the Trainer for ISCB Latin America 2020 |  | Virtual | Mexico | 22-23 October 2020 | Patricia Carvajal-López, Maria Trinidad Bernardi | Virtual | CABANA |
| Train the Trainer for XMXP20 |  | Virtual | Brazil | 44146 | Mindy Stephania Miranda, Adriano Wehrli, Gisele Lopes Nunes, Cesar Augusto Prada-Medina | Virtual | CABANA |
| ISCB Latin America Conference: Train the Trainer Workshop | Universidad Andrés Bello | Vina del Mar | Chile | 5th November 2018 | Cath Brooksbank, Selene Fernandez-Valverde, Alejandro Reyes-Muñoz, Piv Gopalasingam | In-person | CABANA |
| Train the Trainer at UniAndes | Universidad de los Andes | Bogota | Colombia | 15-16 November 2018 | Sarah Morgan, Piv Gopalasingam | In-person | CABANA |
| Train the Trainer at ITV | Instituto Tecnologico Vale | Belem | Brazil | 29-30 August 2019 | Piv Gopalasingam | In-person | CABANA |
| Train the Trainer at USMP | Universidad San Martín de Porres | Lima | Peru | 14-15 October 2019 | Ricardo Fujita | In-person | CABANA |
| Train the Trainer at EMBL-EBI for secondee cohort 1 | EMBL-EBI | Hinxton | UK | 24-25 January 2019 | Sarah Morgan, Piv Gopalasingam | In-person | CABANA |
| Train the Trainer at EMBL-EBI for secondee cohort 2 | EMBL-EBI | Hinxton | UK | 19-20 November 2019 | Sarah Morgan, Piv Gopalasingam | In-person | CABANA |
| Train the Trainer for A2B2C conference | Universidad de Buenos Aires | Virtual | Argentina | 44501 | Carlos Pablo Modenutti, Maria Bernardi | Virtual | Supported |
| Sustainable food production (Plants) | Universidad de Costa Rica | Virtual | Costa Rica | 13-17 December 2021 | Andres Gatica-Arias, Alexandra Holinski | Virtual | CABANA |
| Communicable diseases | Universidad Peruana Cayetano Heredia and Universidad San Martin de Porres | Virtual | Peru | 14-18 February 2022 | Pedro Eduardo Romero-Condori, Dayane Rodriguez-Araujo | Virtual | CABANA |
| Biodiversity | Universidade Federal de Minas Gerais and Instituto Tecnologico Vale | Virtual | Brazil | 2-10 August 2021 | Francisco Pereira-Lobo | Virtual | CABANA |
| Livestock genomics | EMBL-EBI | Hinxton | UK | 1-5 April 2019 | Cath Brooksbank | In-person | CABANA |

# 5. Supplementary Data 5. Summary of workshops delivered by the CABANA project

| **Title** | **Institution** | **Location** | **Country** | **Dates** | **Hosts** | **CABANA hosts** | **Format** | **CABANA or supported?** | **Challenge area** |
| --- | --- | --- | --- | --- | --- | --- | --- | --- | --- |
| Exploring biological networks for disease applications | FIOCRUZ | Salvador, Bahia | Brazil | 8-12 Nov 2021 | Dario Fernandez do Porto, Henning Hermjakob, Pablo Ivan Pereira-Ramos | Adrian Turjanski | Virtual | CABANA | Communicable diseases |
| Genome Analysis using the web-based platform "Galaxy" | INTA | Buenos Aires | Argentina | 7-8 Nov 2021 | Maximo Lisandro Rivarola | Maximo Lisandro Rivarola | Virtual | CABANA | Communicable diseases |
| Curso Internacional: Estrategias bioinformáticas para el estudio de enfermedades tropicales desatendidas | Uni de. Antioquia | Medellin | Colombia | 21-25 Jun 2021 | Dario Fernandez do Porto, Rodrigo Alonso Ochoa Deossa | Marco Aurelio Cristancho/ Alejandro Reyes-Muñoz | Virtual | Supported | Communicable diseases |
| Applied bioinformatics, molecular epidemiology, and the SARS-CoV-2 Pandemic | Universidad de los Andes | Bogotá | Colombia | 1-4 Dec 2020 | Marco Aurelio Cristancho | Marco Aurelio Cristancho | Virtual | CABANA | Communicable diseases |
| Metagenomics and QIIME2 | ITV | Belem, Para | Brazil | 5-9 Oct 2020 | Greg Caporaso | Guilherme Oliveira | Virtual | Supported | Communicable diseases |
| CABANA International Symposium: the genome of SARS-CoV-2, its evolution and epidemiology in Latin America | CIP | Lima | Peru | 1-2 Oct 2020 | Jan Kreuze, Ricardo Fujita | Jan Kreuze | Virtual | CABANA | Communicable diseases |
| Applications of Next-Generation Sequencing to the study of Emerging and Neglected human and animal diseases | IABIMO, INTA | Buenos Aires | Argentina | 2-6 Mar 2020 | Marisa Farber | Maximo Lisandro Rivarola | In-person | Supported | Communicable diseases |
| Structural Bioinformatics | Universidad Nacional de Cuyo | Mendoza | Argentina | 7-8 Nov 2019 | Adrian Turjanski / Carlos Modenutti | Adrian Turjanski / Carlos Modenutti | In-person | CABANA | Communicable diseases |
| Introduction to Chemoinformatics for Drug Discovery | CINVESTAV | Mexico City | Mexico | 15-18 Oct 2019 | Fabien Plisson, Jose Medina-Franco | Fabien Plisson | In-person | CABANA | Communicable diseases |
| Latin American workshop in Structural Bioinformatics of Proteins | Universidad de Antioquia | Medellin | Colombia | 23-27 Sept 2019 | Rodrigo Alonso Ochoa Deossa, Carlos Enrique Muskus | Marco Aurelio Cristancho | In-person | Supported | Communicable diseases |
| FCEN- UBA Functional genomics and chemical biology resources | FCEN-UBA | Buenos Aires | Argentina | 15-16 Oct 2018 | Adrian Turjanski, Carlos Modenutti | Adrian Turjanski, Carlos Modenutti | In-person | CABANA | Communicable diseases |
| Managing a bioinformatics core facility | Uni. Costa Rica | San Jose | Costa Rica | 1-3 Dec 2021 | Rebeca Campos-Sánchez, Maripaz Montero-Vargas, Nicole M. Scherer, Thiago Parente | Rebeca Campos-Sánchez | Virtual | CABANA | Cross-domain |
| Metagenomics for viruses | FIOCRUZ | Manaus | Brazil | 8-12 Nov 2021 | Arthur Gruber | Guilherme Oliveira | Virtual | CABANA | Cross-domain |
| Intro to R and BioConductor | INTA | Buenos Aires | Argentina | 8-9 Nov 2021 | Maximo Lisandro Rivarola / Cei Abreu-Goodger | Maximo Lisandro Rivarola / Cei Abreu-Goodger | Virtual | CABANA | Cross-domain |
| Policy symposium | CIP | Lima | Peru | 15/10/2019 | Jan Kreuze, Hannele Lindqvist-Kreuze, Noelle L. Anglin, Ricardo Fujita | Jan Kreuze, Hannele Lindqvist-Kreuze, Noelle L. Anglin, Ricardo Fujita | In-person | CABANA | Cross-domain |
| RNAseq and network analysis in Plant Genomics | IBAM, Mendoza | Mendoza | Argentina | 6-9 Nov 2019 | Maximo Lisandro Rivarola | Maximo Lisandro Rivarola | In-person | CABANA | Cross-domain |
| Metagenomics | UPCH | Lima | Peru | 15/07/2019 | Jan Kreuze/Ricardo Fujita / Sociedad Peruana SPBBM / Daniel Guerra-Giraldez | Ricardo Fujita | In-person | Supported | Cross-domain |
| Introduction to R and Bioconductor for genomic analyses | Langebio CINVESTAV | Irapuato | Mexico | 20-24 May 2019 | Cei Abreu-Goodger | Cei Abreu-Goodger | In-person | CABANA | Cross-domain |
| Introduction to Metagenomics | FCEN-UBA | Buenos Aires | Argentina | 2-6 Dec 2019 | Adrián Turjanski, Marcelo Adrián Martí, Carlos Modenutti, Alejandro Reyes-Muñoz | Adrián Turjanski, Marcelo Adrián Martí, Carlos Modenutti, Alejandro Reyes-Muñoz | In-person | CABANA | Protection of Biodiversity |
| Biodiversity symposium | ITV | Belem | Brazil | 2-4 Sept 2019 | Guilherme Oliveira | Guilherme Oliveira | In-person | CABANA | Protection of Biodiversity |
| Exploring Biodiversity through Bioinformatics | CIP | Lima | Peru | 9-12 Apr 2019 | Jan Kreuze, Noelle L. Anglin | Jan Kreuze, Noelle L. Anglin | In-person | CABANA | Protection of Biodiversity |
| Langebio CINVESTAV Introduction to NGS workshop | Langebio CINVESTAV | Irapuato | Mexico | 18-20 Mar 2019 | Selene Fernandez-Valverde, Alfredo Heriberto Herrera-Estrella | Selene Fernandez-Valverde, Alfredo Heriberto Herrera-Estrella | In-person | CABANA | Protection of Biodiversity |
| Metagenomics Workshop | ULA | Bogotá | Colombia | 3-6 Dec 2018 | Alejandro Reyes-Muñoz | Alejandro Reyes-Muñoz | In-person | CABANA | Protection of Biodiversity |
| Analysis of Crop Genomics Data | Universidad de los Andes | Bogotá | Colombia | 1-11 Mar 2021 | Marco Aurelio Cristancho | Marco Aurelio Cristancho | Virtual | CABANA | Sustainable food production |
| NGS applied to virome sequencing in agricultural systems - computational training | UCR | San Jose | Costa Rica | 24 Feb - 4 Mar 2020 | Rebeca Campos-Sanchez, Andres Gatica-Arias | Rebeca Campos-Sanchez, Andres Gatica-Arias | In-person | CABANA | Sustainable food production |
| Genotyping by Sequencing Applied to Biodiversity Analyses and Plant Breeding | UCR | San Jose | Costa Rica | 6-9 Aug 2019 | Rebeca Campos-Sanchez, Andres Gatica-Arias, César Rodríguez-Sanchez | Rebeca Campos-Sanchez, Andres Gatica-Arias, César Rodríguez-Sanchez | In-person | CABANA | Sustainable food production |
| Genomic Analysis of Crop Biodiversity using R | Langebio CINVESTAV | Irapuato | Mexico | 25-28 Jun 2019 | Octavio Martinez de la Vega, Humberto Reyes-Valdes | Octavio Martinez de la Vega | In-person | CABANA | Sustainable food production |
| INTA Functional Genomics | INTA | Buenos Aires | Argentina | 18-19 Oct 2018 | Maximo Lisandro Rivarola | Maximo Lisandro Rivarola | In-person | CABANA | Sustainable food production |

# 6. Supplementary Data 6. Details of research projects developed as part of the CABANA project

| **Project title** | **Lead investigator (institution, country)** | **Collaborator institutions** | **Budget (sterling pounds £)** | **Publications** |
| --- | --- | --- | --- | --- |
| Whole Genome Sequencing of Race II and Low-Coverage Sequencing of Twelve Additional Isolates of the Coffee Rust, Hemileia Vastatrix | Marco Cristancho (Universidad de los Andes, Colombia) | CINVESTAV, Mexico  Vale Institute, Brazil  CIP, Peru | 30,000 | Manuscript in preparation |
| Analysis of natural and induced genetic diversity of populations of coffee in Costa Rica and Mexico using GBS | Andrés Gatica-Arias (Universidad de Costa Rica, Costa Rica) | CINVESTAV, Mexico | 29,500 | Till, B. J., Jiménez-Madrigal, J. P., and Gatica-Arias, A. (2024). Plant Functional Genomics, Methods and Protocols, Volume 1. Methods Mol. Biol. 2787, 123–139. doi: 10.1007/978-1-0716-3778-4_8  Jiménez-Madrigal, J. P., Till, B. J., and Gatica-Arias, A. (2024). Plant Functional Genomics, Methods and Protocols, Volume 1. Methods Mol. Biol. 2787, 107–122. doi: 10.1007/978-1-0716-3778-4_7 |
| The SARS-CoV-2 genome, its evolution and epidemiology in Latin America | Alfredo Heriberto Herrera-Estrella (CINVESTAV, Mexico) | Universidad de los Andes, Colombia  CIP, Peru  FCEN-UBA, Argentina  UCR, Costa Rica  Vale Institute, Brazil | 102,000 | Molina-Mora, J. A., Reales-González, J., Camacho, E., Duarte-Martínez, F., Tsukayama, P., Soto-Garita, C., et al. (2023). Overview of the SARS-CoV-2 genotypes circulating in Latin America during 2021. Frontiers Public Heal 11, 1095202. doi: 10.3389/fpubh.2023.1095202 |
| The Genome of Yerba Mate (Ilex Paraguariensis) | Carlos Modenutti (Universidad de Buenos Aires, Argentina) | Vale Institute, Brazil | 29,000 | Vignale, F. A., Garcia, A. H., Modenutti, C. P., Sosa, E. J., Defelipe, L. A., Oliveira, R. R. M., et al. (2024). Yerba mate (Ilex paraguariensis) genome provides new insights into convergent evolution of caffeine biosynthesis. bioRxiv, 2023.09.08.556846. doi: 10.1101/2023.09.08.556846 |
| Paleometagenomics of Microbialites in the Puna | Adrián Turjanski (Universidad de Buenos Aires, Argentina) | USMP, Peru | 25,000 | Manuscript in preparation |
| Exploring the microbiomes of soils associated with contrasting agricultural systems across Latin America | Jan Kreuze (CIP, Peru) | CNIA-INTA, Argentina  Uniandes, Colombia | 30,000 | Gómez-Godínez, L. J., Ochoa, V., Faggioli, V., and Cristancho, M. (2024). Exploring the soil associated bacterial microbiome of coffee plantations in different regions of Colombia: a metabarcoding approach. Trop. Subtrop. Agroecosystems 27. doi: 10.56369/tsaes.5196  Ochoa-Henriquez, V. H., Faggioli, V., Gómez-Godínez, L. J., Rivarola, M., and Cristancho, M. (2024). Colombian coffee (Coffea arabica L.) plantations: a taxonomic and functional survey of soil fungi. Front. Sustain. Food Syst. 8, 1345383. doi: 10.3389/fsufs.2024.1345383 |
| Genomic Diversity of Yeasts Associated to Natural Agave Fermentation | Cei Abreu-Goodger (CINVESTAV, Mexico) | FCEN-UBA, Argentina | 27,400 | Cabrera-Toledo, D., Mendoza-Galindo, E., Larranaga, N., Herrera-Estrella, A., Vásquez-Cruz, M., and Hernández-Hernández, T. (2022). Genomic and Morphological Differentiation of Spirit Producing Agave angustifolia Traditional Landraces Cultivated in Jalisco, Mexico. Plants 11, 2274. doi: 10.3390/plants11172274 |
